# Modular chromosome rearrangements reveal parallel and nonparallel adaptation in a marine fish

**DOI:** 10.1002/ece3.5828

**Published:** 2020-01-11

**Authors:** Tony Kess, Paul Bentzen, Sarah J. Lehnert, Emma V. A. Sylvester, Sigbjørn Lien, Matthew P. Kent, Marion Sinclair‐Waters, Corey Morris, Brendan Wringe, Robert Fairweather, Ian R. Bradbury

**Affiliations:** ^1^ Fisheries and Oceans Canada Northwest Atlantic Fisheries Centre St. John's NL Canada; ^2^ Biology Department Dalhousie University Halifax NS Canada; ^3^ Department of Animal and Aquacultural Sciences Faculty of Biosciences Centre for Integrative Genetics Norwegian University of Life Sciences Ås Norway; ^4^ Organismal and Evolutionary Biology Research Programme University of Helsinki Helsinki Finland; ^5^ Fisheries and Oceans Canada Bedford Institute of Oceanography Dartmouth NS Canada

**Keywords:** Atlantic cod, environmental association, genomic architecture, marine, migration, parallel evolution

## Abstract

Genomic architecture and standing variation can play a key role in ecological adaptation and contribute to the predictability of evolution. In Atlantic cod (*Gadus morhua*), four large chromosomal rearrangements have been associated with ecological gradients and migratory behavior in regional analyses. However, the degree of parallelism, the extent of independent inheritance, and functional distinctiveness of these rearrangements remain poorly understood. Here, we use a 12K single nucleotide polymorphism (SNP) array to demonstrate extensive individual variation in rearrangement genotype within populations across the species range, suggesting that local adaptation to fine‐scale ecological variation is enabled by rearrangements with independent inheritance. Our results demonstrate significant association of rearrangements with migration phenotype and environmental gradients across the species range. Individual rearrangements exhibit functional modularity, but also contain loci showing multiple environmental associations. Clustering in genetic distance trees and reduced differentiation within rearrangements across the species range are consistent with shared variation as a source of contemporary adaptive diversity in Atlantic cod. Conversely, we also find that haplotypes in the LG12 and LG1 rearranged region have diverged across the Atlantic, despite consistent environmental associations. Exchange of these structurally variable genomic regions, as well as local selective pressures, has likely facilitated individual diversity within Atlantic cod stocks. Our results highlight the importance of genomic architecture and standing variation in enabling fine‐scale adaptation in marine species.

## Graphical abstract

Understanding parallel evolution in wild populations has long been central to evolutionary biology; genomic evidence has recently indicated an important role for chromosomal structural variation in parallel adaptation. Atlantic cod show repeated evidence of local adaptation across diverse marine habitats, with recent evidence implicating the involvement of a series of chromosomal rearrangements. We explore the distribution and trait and environmental associations of these chromosomal rearrangements within populations range‐wide. We find that across the North Atlantic contemporary adaptation and individual‐based biocomplexity in Atlantic cod is frequently underlain by shared chromosomal rearrangements.

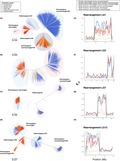

## 1 INTRODUCTION

Instances of parallel evolution provide the opportunity to quantify the variety of solutions to ecological problems at different scales of organization, from molecular to phenotypic levels. Understanding the processes underlying parallel genomic adaptation is a fundamental goal in biology and can clarify to what extent evolution is deterministic (Blount, Lenski, & Losos, 2018; Conte, Arnegard, Peichel, & Schluter, 2012; Elmer & Meyer, 2011; Stern, 2013). Genomic organization and history may be integral to genomic parallelism, affecting the rate and predictability of adaptation (Stern, 2013; Tigano & Friesen, 2016). Recent evidence has demonstrated that the amount of shared variation within a genome and how this variation is organized structurally can facilitate local adaptation and may influence rates of genomic parallelism (Nelson & Cresko, 2018; Pearse, Miller, Abadia‐Cardoso, & Garza, 2014). However, empirical support demonstrating the role of shared variation and structural variation across systems is only beginning to be understood.

Genomic architecture, the organization and interactions of genes within the genome, can affect rates of adaptation (Flaxman, Wacholder, Feder, & Nosil, 2014; Rogers, Mee, & Bowles, 2013). Genomic rearrangements, such as inversions, can reduce recombination within rearranged genomic regions, allowing evolution of co‐adapted complexes of alleles advantageous for adaptation in heterogeneous environments (Barb et al., 2014; Hoffmann & Rieseberg, 2008; Kirkpatrick & Barton, 2006). Compartmentalization of genes underlying different traits into functionally independent units (i.e., functional modularity) may also affect rates of adaptation (Ragland et al., 2017; Rogers et al., 2013; Stern & Orgogozo, 2007; Wagner, Pavlicev, & Cheverud, 2007) and can enable evolution of phenotypes adapted to different selective pressures without fitness reduction caused by genetic constraints (Wagner et al., 2007).

Standing variation in these genomic architectural features can allow rapid responses to selection and directly influence genomic parallelism (Bolnick, Barrett, Oke, Rennison, & Stuart, 2018). Examples of repeated incorporation of shared rearrangements into the genetic architecture of adaptive traits have been identified across taxa, including loci contributing to crypsis in *Timema cristinae* stick insects (Lindtke et al., 2017), diapause timing in apple maggot flies (*Rhagoletis pomonella*, Feder et al., 2003; Ragland et al., 2017), and rapid colonization of novel habitats in threespine sticklebacks (*Gasterosteus aculeatus*, Bassham, Catchen, Lescak, Hippel, & Cresko, 2018; Jones et al., 2012). However, understanding the eco‐evolutionary functions of genomic architectural features has only recently become possible with high‐resolution sequencing, genomic mapping, and genome assemblies (Wellenreuther & Bernatchez, 2018).

Atlantic cod (*Gadus morhua*), a heavily exploited, demersal, broadcast spawning marine fish, provides a study system well suited to investigate the extent of modularity and parallelism among genomic architectural features. Atlantic cod exhibit high connectivity and dispersal, but also show divergent adaptation and genetic structuring associated with genomic architectural variation (Barth et al., 2017; Sinclair‐Waters, Bentzen, et al., 2018). Past genetic studies of Atlantic cod have suggested parallel environmental adaptation to temperature and salinity in the east and west Atlantic (Berg et al., 2015; Bradbury et al., 2010). Phenotypic parallelism of migratory and stationary ecotypes occurring in Europe and North American coastal waters has also been identified (Robichaud & Rose, 2004). This environmental and behavioral divergence has likely been facilitated by rearrangements consisting of multiple potential inversions on four separate linkage groups (LG 1, 2, 7, and 12, Kirubakaran et al., 2016; Sodeland et al., 2016). However, several outstanding questions remain regarding the origin and function of diverged, rearranged genomic regions among individual Atlantic cod across the range. The extent of coinheritance and functional modularity of rearrangements in Atlantic cod, as well as the extent that the same rearrangements are shared across the Atlantic remains unclear. Previous range‐wide comparisons have revealed interchromosomal linkage between rearranged regions, suggesting a shared evolutionary trajectory for rearranged regions (Berg et al., 2015; Bradbury et al., 2014). However, structuring associated with environmental clines and trans‐Atlantic divergence can produce interchromosomal linkage that may mask how frequently chromosomal rearrangements are co‐inherited at finer scales.

Here, we build on previous studies and explore the potential for exchange of these rearrangements across the entire Atlantic range, and their role in local adaptation to fine‐scale ecological variation in Atlantic cod. First, we quantify coinheritance of chromosomal rearrangements within populations to test whether multiple rearrangements show a shared evolutionary trajectory. We then conduct environmental and migratory associations with genomic markers using multivariate and machine‐learning methods that can detect relationships between alleles that would be missed in single‐locus models and genome scans (Forester, Lasky, Wagner, & Urban, 2018; Gagnaire & Gaggiotti, 2016; Stephan, Stegle, & Beyer, 2015). Next, we use multivariate clustering to determine whether loci within rearrangements show consistent associations with the same environmental variables, indicating functional modularity (Lotterhos, Yeaman, Degner, Aitken, & Hodgins, 2018). Finally, we test for signatures of adaptation from shared variation to infer whether the same haplotypes in rearranged regions are shared across the Atlantic. Overall, the results from our study highlight how features of genomic architecture and history can enable repeated differentiation and adaptation across a variety of habitats within a highly connected marine species.

## 2 METHODS

### 2.1 Genotype data

**Figure 1:**
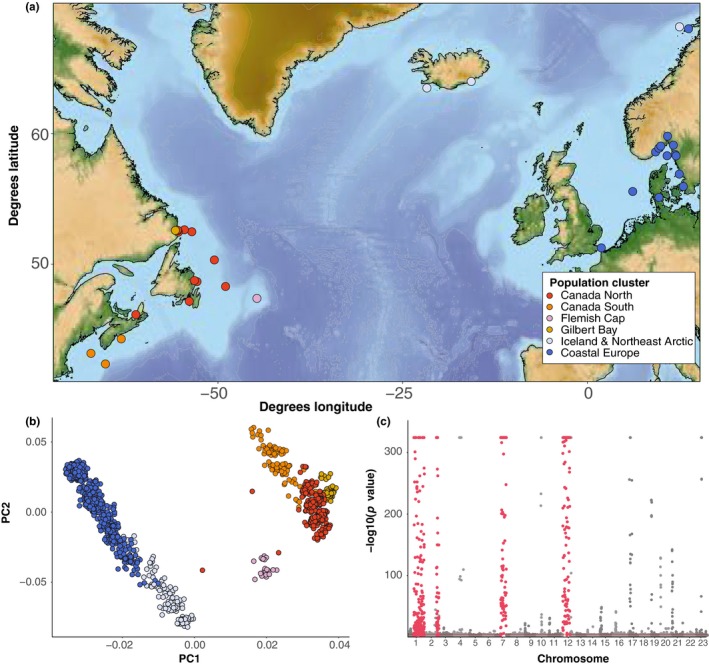
Geographic location of sampling sites, population clusters, and single nucleotide polymorphism (SNP) correlations with principal component axes associated with population structure in Atlantic cod. (a) Location and population clusters of sampling sites. (b) Population clustering of samples on the first two principal component axes in *pcadapt*. (c) Significance of correlation of each SNP (*p* value) with the *K* = 25 retained principal components in *pcadapt*. Chromosome rearrangements are marked in red

To identify population structure and genome architecture variation across the range of Atlantic cod, we combined five datasets that were previously genotyped using the same 12K SNP array, along with additional samples from the Flemish Cap genotyped using the same array (Barth et al., 2017; Berg et al., 2017; Kess et al., 2019; Sinclair‐Waters, Bentzen, et al., 2018; Sinclair‐Waters, Bradbury, et al., 2018). Atlantic cod genotypes from North America and Europe were obtained from 44 separate sampling trips from 37 distinct sampling locations (Figure 1a, sampling site and year in Table S1). Detailed genotyping and collection information are available in the studies cited above. These genotypes were combined into a final dataset of *n* = 1,230 (*n* = 531 North America, *n* = 699 Europe) individuals genotyped at 6,669 informative SNPs genotyped in all populations, corrected for strand flips, and filtered to remove SNPs with ambiguous genotype coding across datasets.

### 2.2 Range‐wide population structure

We quantified range‐wide population structure using principal component analysis (PCA) and t‐distributed stochastic neighbor embedding (t‐SNE), a nonlinear machine‐learning clustering algorithm (van der Maaten & Hinton, 2008), with genotypes from all 1,230 individuals from all populations. We detected outliers driving population clustering in PCA by calculating Mahalanobis distances for each SNP from a vector of *z*‐scores describing its relationship with *K* tested principal components. Using the R package *pcadapt* (Luu, Bazin, & Blum, 2017), we carried out PCA with *K* = 2 to identify SNPs associated with the first two PC axes explaining most geographic variation, assuming a false discovery rate (FDR, Storey & Tibshirani, 2003) cutoff of *q* = 0.1. We then used *K* = 25 to identify SNPs that were correlated with principal components that explained less variation but captured more subtle genetic differences between populations and individuals. To represent this variation on two axes, we also calculated t‐SNE scores for each individual using the *tSNE* R package (Donaldson, 2016). We assigned a perplexity value of 25; this parameter characterizes the number of effective nearest neighbors expected in the data.

### 2.3 Individual genetic variation

To quantify individual genetic variation, we separated individuals into geographic groups from Canada North, Canada South, Gilbert Bay, Flemish Cap, Iceland and Northeast Artic, and Coastal Europe (Figure 1a,b). These groupings are supported by separation of geographic regions in both PCA and t‐SNE, although the split with Glibert Bay is more distinct in t‐SNE, suggesting multiple axes of genetic divergence (Figures 1b and S1). We then conducted PCA within each of these clusters using *pcadapt*. Outliers differentiated between individuals were identified by significance of Mahalanobis distances for each SNP, using an FDR of *q* = 0.1. We set *K* = 25 to increase the chance of identifying SNPs making marginal contributions to individual structuring. PCA clustering in the Canada North region has been performed previously in Kess et al., 2019, but was limited to *K* = 1 principal components and did not include the population in the Gulf of St. Lawrence (CSG), as the goal of the previous study was to identify the primary driver of individual genetic structuring within the Northern cod stock around Newfoundland and Labrador.

### 2.4 Range‐wide genetic structure of rearrangements

Separate Bayesian clustering of SNPs within each chromosomal rearrangement was conducted using STRUCTURE v2.3.4 (Pritchard, Stephens, & Donnelly, 2000) to determine individual rearrangement genotype. We carried out separate analyses of the 531 individuals within North America and the 699 individuals within Europe. We conducted separate runs for all SNPS within rearrangements on LG1, LG2, LG7, and LG12 in R using *parallelstructure* (Besnier & Glover, 2013). For each rearrangement, we ran three replicate Markov Chain Monte Carlo runs of 100,000 burn‐ins followed by 500,000 iterations, assuming *K* = 2. Bayesian clustering of the LG1 rearranged region was previously carried out by Kess et al., 2019, but did not include the Flemish Cap samples. Individual genetic ancestry proportions were summarized and visualized using CLUMPAK (Kopelman, Mayzel, Jakobsson, Rosenberg, & Mayrose, 2015). Filtering of rearrangement SNPs based on genome locations used by Berg et al., 2017 was performed using *genepopedit* (Stanley, Jeffery, Wringe, DiBacco, & Bradbury, 2016), and conversion to STRUCTURE format was carried out with PGDSpider (Lischer & Excoffier, 2012)_._ We calculated rearrangement frequencies by grouping individual rearrangement genotypes into homozygous rearranged, homozygous nonrearranged, and heterozygous categories inferred from STRUCTURE admixture proportions (*Q*‐values). Because categorization of chromosomal orientations into rearranged and nonrearranged categories requires phylogenetic comparison across multiple species to test the origin of rearranged regions, we use “rearranged” genotype and “nonrearranged” genotype designations only to characterize alternative chromosomal states, but follow designations used by Berg et al. (2017).

### 2.5 Coinheritance and linkage disequilibrium

We tested for coinheritance of all *pcadapt* outlier SNPs identified within each regional cluster exhibiting within‐population architectural variation using two linkage‐based methods. We calculated an *r* ^2^ matrix quantifying pairwise linkage disequilibrium (LD) between each SNP using plink 1.9 (Chang et al., 2015). LD calculations were performed separately within Canada North (*n* = 553 SNPs), Canada South (*n* = 490 SNPs), Iceland and Northeast Arctic (*n* = 618 SNPs), and Coastal Europe population clusters (*n* = 730 SNPs). We visualized linkage between outlier SNPs using an LD heatmap in the *superheat* R package (Barter & Yu, 2017). Using the *LDna* R package (Kemppainen et al., 2015), we generated network plots to visualize each SNP as a node connected by edges exceeding an *r* ^2^ threshold of .25.

### 2.6 Environmental and migratory association with genomic variation

To identify association of all 6,669 SNPs with environmental gradients, we carried out genome‐wide association using two polygenic methods: redundancy analysis (RDA, Rao, 1964) and random forest (Breiman, 2001). We conducted RDA and random forest separately for all individuals in North America and in Europe to limit the possibility of nonparallel genetic architecture and residual population structure diluting signals of local adaptation. We extracted 12 environmental variables for each sampling location from the Bio‐ORACLE v2.0 database (Assis et al., 2018) using the *sdmpredictors* R package (Bosch, 2018). Environmental variables were obtained from mean bottom depth layers for monthly (2000–2014) mean, minimum and maximum values for salinity, dissolved oxygen, and temperature. To reduce the number of correlated variables in RDA, we standardized each variable by its standard deviation and then conducted separate PCAs on variables for salinity, temperature, and dissolved oxygen. We used the first PC for each of these variables as individual phenotypes used as predictors in an RDA model to identify environmental association. SNPs with absolute RDA scores exceeding the 95th percentile were categorized as outliers. We conducted redundancy analysis using the *vegan* R package (Oksanen et al., 2011). We did not include a covariate for geographic structure in environmental association, as true signals of association may be lost when environmental adaptation tracks geographic separation (Yeaman et al., 2016), and dimensionality reduction used in redundancy analysis has been shown to effectively control for background structure (Forester, Jones, Joost, Landguth, & Lasky, 2016).

We carried out separate random forest regressions for each environmental variable in North America and Europe. Loci significantly associated with each environmental variable were identified as those with mean decrease in accuracy (MDA) scores exceeding a threshold identified by MDA drop‐off inferred from binning of MDA scores. SNPs with MDA values below this threshold exhibited low power in predicting environmental features. Random forest regressions were conducted using the *randomForest* R package (Liaw & Wiener, 2002).

We explored genetic associations with migration phenotype for all individuals in Europe using all 6,669 SNPs, following the association methods used in Kess et al. (2019) in North America. Regional‐level migration phenotype was assigned to each individual from migratory classes identified by Robichaud and Rose (2004). As above, we identified SNP associations with migratory phenotype using redundancy analysis without correction for geographic structure and random forest classification.

To determine the direction of association between environmental and migratory variables and rearrangement frequencies, we calculated Pearson's correlation between the frequency of each rearrangement and each environmental variable. We assessed significance at a lenient *p* < .05, as the goal of this test was to quantify direction of association.

### 2.7 Clustering of loci by multiple environmental associations

We examined patterns of SNP co‐association to uncover groups of loci that exhibited similar patterns of associations across multiple environmental variables. As in Lotterhos et al. (2018), we calculated absolute values of Pearson's correlation coefficient (*r* ^2^) between each SNP and all environmental variables: principal component, minimum, maximum and mean values for salinity, temperature, and dissolved oxygen. We calculated separate correlations for each SNP that was identified as significantly associated with environment in RDA in Europe and in North America. We then produced a separate pairwise Euclidean distance matrix for Europe and North America from distances between SNPs and their associations across variables and carried out hierarchical clustering on each matrix using Ward's hierarchical clustering algorithm in R using the *hclust* function. Loci that exhibit shared patterns of association across multiple environmental variables will be found in the same cluster using hierarchical clustering; these clusters represent proxies for functional modules. The R package *superheat* was then used to visualize patterns of shared associations using paired dendograms and heatmaps.

### 2.8 Signatures of adaptation from shared variation

To identify whether environment and migration‐associated rearrangements represent adaptation from shared variation, or from de novo mutations, we measured differentiation and separation in trees of genetic distance between European and North American individuals with shared rearrangement genotypes. We compared *F*_ST_ and *d_XY_* across linkage groups LG1, LG2, LG7, and LG12 between individuals with each rearrangement genotype in North America and Europe and calculated nucleotide diversity (*π*) separately for each rearrangement genotype in North America and Europe. *F*_ST_ calculations were carried out in the *diveRsity* R package (Keenan, McGinnity, Cross, Crozier, & Prodöhl, 2013). We used Beagle 5.0 (Browning, Zhou, & Browning, 2018) to phase vcf files and then calculated *d_XY_*, and *π* using the R package *PopGenome* (Pfeifer, Wittelsbürger, Ramos‐Onsins, & Lercher, 2014). Differences in *F*_ST_, *d_XY_*, and *π* between rearrangement genotype groups, and within and outside structurally variable regions on each chromosome were tested using a Mann–Whitney *U* test (Mann & Whitney, 1947). We then calculated range‐wide individual genetic distance trees for each rearrangement separately with all 1,230 genotyped individuals. We calculated Cavalli‐Szforza and Edwards chord distances (Cavalli‐Sforza & Edwards, 1967) with 1,000 loci bootstraps in POPULATIONS v1.2.33 (Langella, 2012) and visualized trees using FigTree v1.74 (Rambaut, 2012).

## 3 RESULTS

### 3.1 Population structure and individual genetic variation

**Figure 2:**
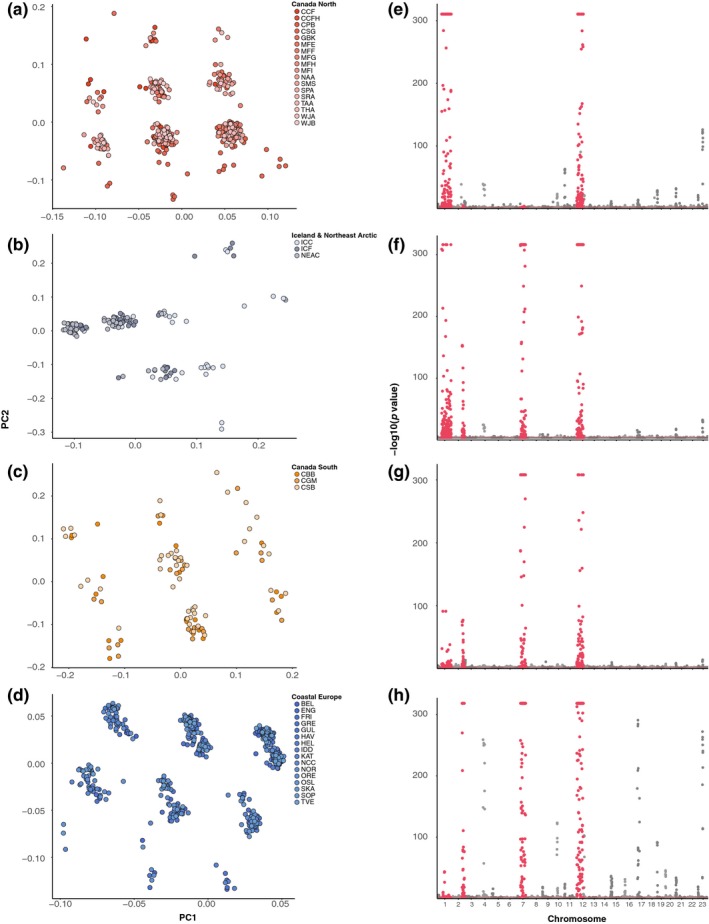
Clustering of individuals from sampling sites within major population genetic clusters and single nucleotide polymorphism (SNP) correlations with principal component axes associated with individual genetic structure within each major cluster. (a–d) Individual clustering of samples on the first two principal component axes in *pcadapt* in Canada North, Iceland and Northeast Arctic, Canada South, and Coastal Europe population clusters. (e–h) Significance of correlation of each SNP (*p* value) with the *K* = 25 retained principal components in *pcadapt* in Canada North, Iceland and Northeast Arctic, Canada South, and Coastal Europe population clusters. Chromosome rearrangements are marked in red

**Figure 3:**
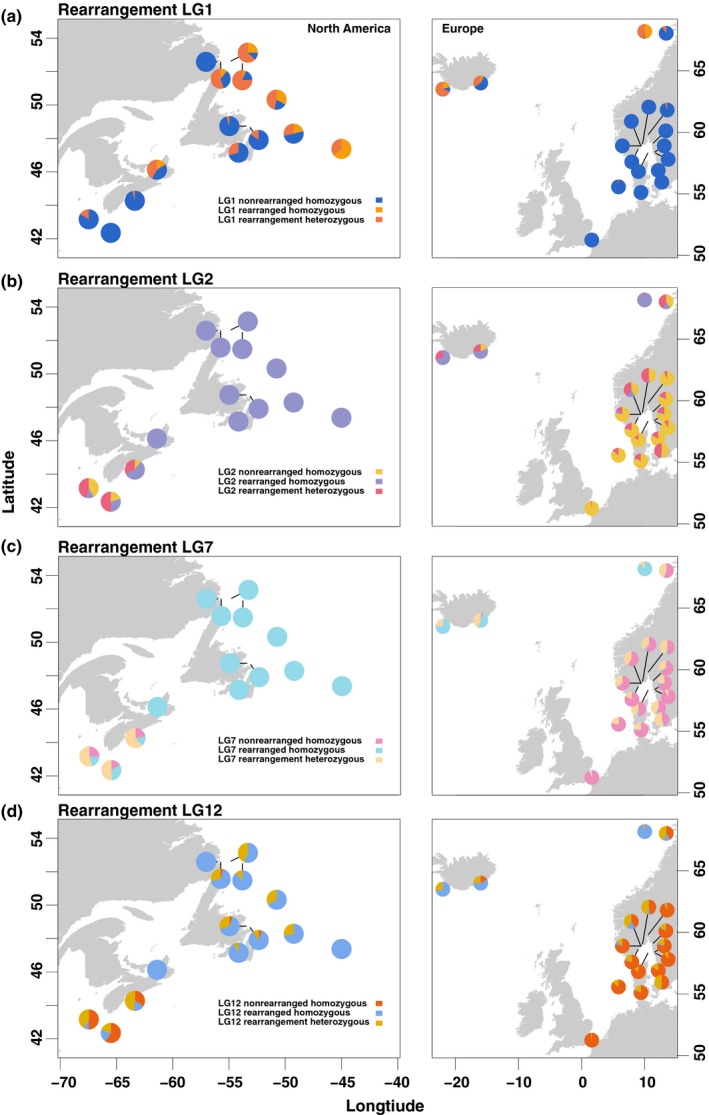
Proportion of homozygous rearranged, homozygous non‐rearranged, and heterozygous individuals for LG1, LG2, LG7, and LG12 rearrangements in sites in North America and Europe. (a) Proportions of LG1 genotypes in North America and Europe (b) Proportions of LG2 genotypes in North America and Europe (c) Proportions of LG7 genotypes in North America and Europe (d) Proportions of LG12 genotypes in North America and Europe

Using dimensionality reduction, we identified population structure associated with differentiation among geographic regions, and structuring by rearrangement genotype within these geographic regions. Structuring within the total dataset uncovered trans‐Atlantic and latitudinal separation (Figures 1b and S1), driven by 1,012 SNPs with significant *q‐*values (<0.1), concentrated in four known rearrangements on LG1, LG2, LG7, and LG12 (Figure 1c). Within the Canada North, Canada South, Iceland and Northeast Arctic, and Coastal Europe regions, we found multiple discrete genetic clusters primarily driven by different complements of chromosomal rearrangements (Figure 2a–d), whereas no clear separation of individuals was observed in the Flemish Cap or Gilbert Bay regions. Clustering along PC1 and PC2 axes corresponded to different combinations of linkage groups with known rearrangements contributing to structuring within each geographic region (Figure 2e–h). Bayesian clustering of LG1, LG2, LG7, and LG12 rearrangements within Europe and North America revealed ancestry proportion distributions consistent with homozygous or heterozygous rearrangement genotypes (*Q* ~ 0, 0.5, 1, Figure S4). We identified the presence of all three genotypes for each rearrangement across several sites in North America and Europe (Figure 3), indicating variation in rearrangement genotype is a consistent driver of individual genetic differences across many Atlantic cod populations (Table S2).

### 3.2 Coinheritance and linkage disequilibrium

**Figure 4:**
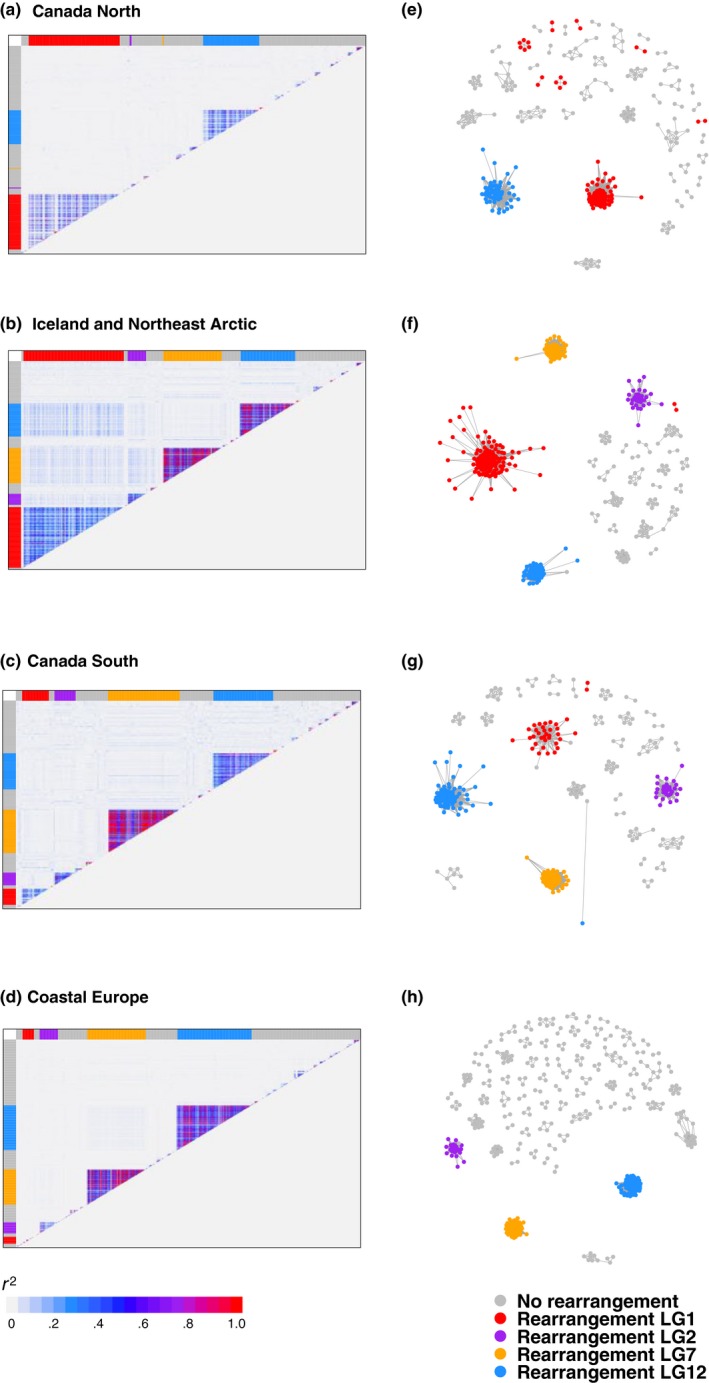
Linkage disequilibrium between outlier single nucleotide polymorphisms (SNPs) significantly correlated with principal component axes describing individual genetic variation in *pcadapt* in Canada North, Iceland and Northeast Arctic, Canada South, and Coastal Europe population clusters. (a–d) Linkage as measured by *r* ^2^ between outlier SNPs visualized using LD heatmaps (e–h) Linkage between outlier SNPs visualized in a linkage network in *LDna* with an *r* ^2^ cutoff of .25. Chromosomal rearrangements are represented by separate colors

Consistent with independent inheritance of rearrangements, we observed low LD of outlier SNPs on different rearrangements within any geographic regions (Figure 4a–d). However, patterns of interchromosomal LD varied slightly between regions, with small elevation in LD observed in Iceland and Northeast Arctic samples relative to other regions (Figure 3). We identified high intrachromosomal LD within each rearrangement, consistent with reduced recombination between alternative rearrangement orientations (Figure 4a–d). Linkage networks also supported extensive LD within rearrangements, and low LD between different rearrangements within each geographic cluster at an *r* ^2^ cutoff of .25, indicating independent inheritance of each rearrangement within each geographic cluster (Figure 4e–h).

### 3.3 Environmental and migratory association with genomic variation

**Figure 5:**
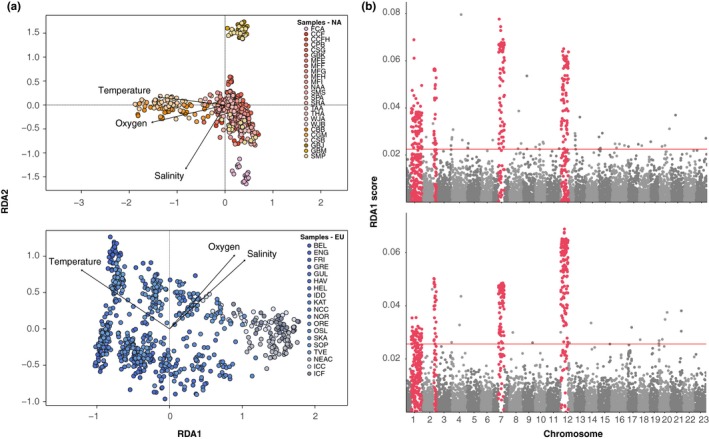
Redundancy analysis of single nucleotide polymorphisms (SNPs) with temperature, salinity, and oxygen in North America and Europe. (a) Biplots of RDA1 and RDA2 scores for each individual color coded by population with vectors describing variation due to oxygen, temperature, and salinity gradients in North America and Europe. (b) Absolute values of scores for each SNP on RDA1 within North America and Europe. Significant SNPs are those with scores greater than the 95th percentile. Chromosome rearrangements are marked in red

Tests of polygenic environmental adaptation revealed associations primarily within chromosomal rearrangements in both North America and Europe, supporting a role for these regions in local adaptation. Redundancy analysis (RDA) indicated that variation in principal components of salinity, oxygen, and temperature was strongly associated in with LG1, LG2, LG7, and LG12 rearrangements in both North America and Europe (Figure 5a,b). Random forest regression of SNPs with environmental variables also revealed consistent association of rearrangements with variation in temperature, oxygen, and salinity (Table 1, Figures S5 and S6). We also identified a shared positive association with at least one temperature variable and LG2, LG7, and LG12 rearrangement haplotype frequency on either side of the Atlantic (Table S3).

**Table 1:** Number of single nucleotide polymorphisms (SNPs) associated with temperature, dissolved O_2_, and salinity in North America and Europe identified using random forest regression, and proportion of environment‐associated SNPs in chromosomal rearrangements

Location	Variable	MDA threshold	Associated SNPs	Proportion of environment ‐associated SNPs in rearrangements
Europe	Maximum temperature	0.05	31	0.81
	Mean temperature	0.02	12	0.92
	Minimum temperature	0.01	22	0.36
	Max dissolved O_2_	1.0	58	0.53
	Mean dissolved O_2_	0.5	27	0.30
	Minimum dissolved O_2_	0.05	81	0.72
	Maximum salinity	0.01	18	0.50
	Mean salinity	0.01	24	0.54
	Minimum salinity	0.01	48	0.57
North America	Maximum temperature	0.05	133	0.67
	Mean temperature	0.01	41	0.95
	Minimum temperature	0.05	43	0.95
	Max dissolved O_2_	10	56	0.95
	Mean dissolved O_2_	10	72	0.97
	Minimum dissolved O_2_	5	83	0.98
	Maximum salinity	0.002	51	0.86
	Mean salinity	0.005	22	0.82
	Minimum salinity	0.01	48	0.79

Redundancy analysis and random forest classification have been previously used to identify an association between migratory behavior and the LG1 rearrangement in North America (Kess et al., 2019). Using both of these methods to repeat this analysis in European waters, we found migratory phenotype associations predominantly with SNPs within the LG1 rearrangement, (Figure S7), suggesting parallelism of genetic architecture for this trait. We also found that frequency of the rearranged LG1 haplotype was positively correlated with migration in both North America and Europe (Table S3).

### 3.4 Clustering of loci by multiple environmental associations

**Figure 6:**
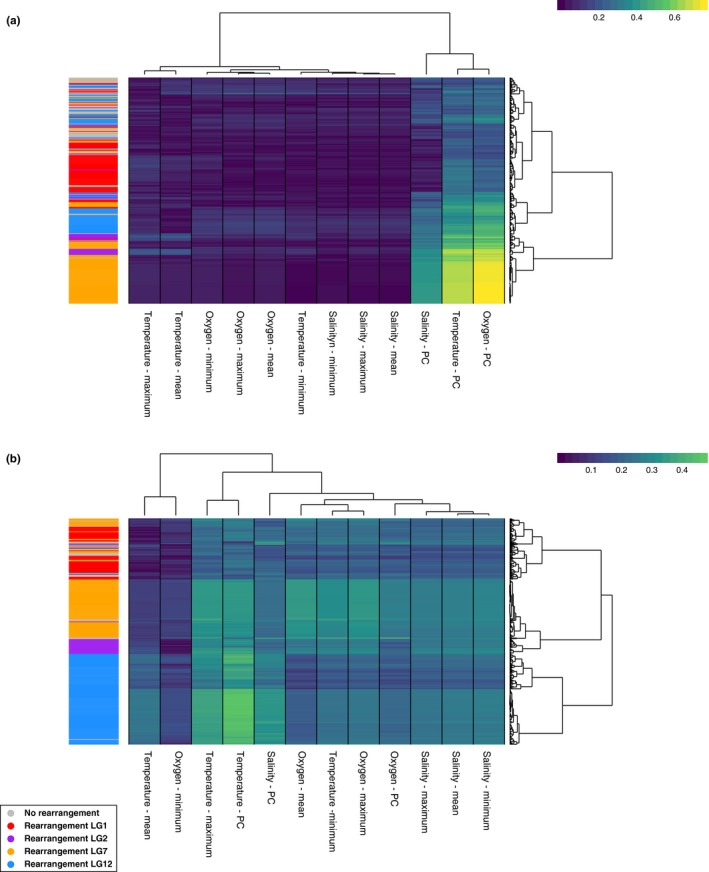
Co‐association of RDA outlier single nucleotide polymorphisms (SNPs) with environmental variables grouped by hierarchical clustering of SNP association across environmental variables in North America (a) and Europe (b)

We found that most SNPs within rearranged regions belonged to the same functional cluster, indicating functional modularity, but in several instances outlier SNPs within multiple rearranged regions were found in the same environmental co‐association cluster (Figure 6a,b). In North America, environmental clusters were driven by strength of association with principal components. Although SNPs within the same rearranged region tended to show similar patterns of co‐association, we observed SNPs from each rearrangement within the same cluster across multiple major co‐association clusters (Figure 6a). In Europe, environmental clustering was driven by associations between principal components as well as individual environmental variables. The pattern of co‐association of SNPs with the same environmental variable across rearrangements was less pronounced in Europe, indicating greater functional modularity. Reduced co‐association between rearrangements was greatest in LG12; SNPs in LG12 exhibited almost no clustering with SNPs within other rearrangements (Figure 6b).

### 3.5 Signatures of adaptation from shared variation

**Figure 7:**
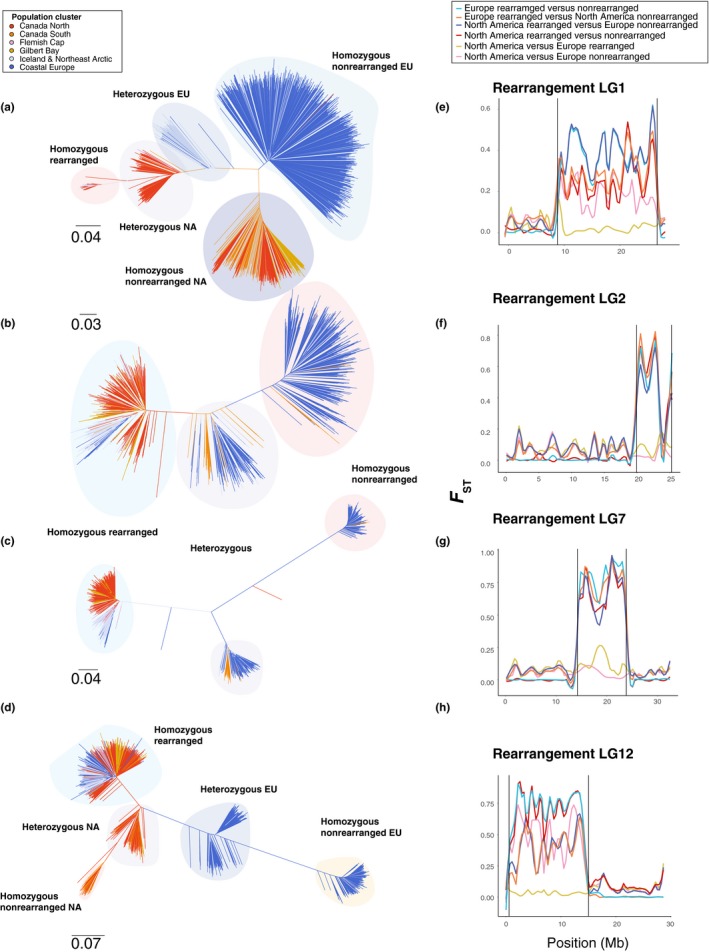
Comparison of *F*_ST_ along LG1, 2, 7, and 12 between individuals with each rearrangement genotype in North America and Europe and genetic distance trees for each rearrangement. (a–d) Comparison of *F*_ST_ along LG1, 2, 7, and 12 between alternative homozygous rearrangement genotype categories in North America and Europe. Black vertical lines indicate the boundaries of each rearrangement. (e–h) Range‐wide individual trees of genetic distance for LG1, 2, 7, and 12 rearrangements calculated using Cavalli‐Szforza and Edwards chord distances

For each rearrangement, trees of genetic distance showed groupings distinct to rearrangement genotype. For LG1, individual genetic distance trees revealed no separation of LG1 homozygous rearranged genotype individuals by geography, supporting a hypothesis of adaptation from shared variation for these rearrangements (Figure 7a). Distinct clusters were identified for European and North American nonrearranged genotype and heterozygous genotype individuals, indicating high trans‐Atlantic differentiation for the nonrearranged LG1 orientation.

In LG2 and LG7, we observed separate clusters corresponding to homozygous rearranged, homozygous nonrearranged, and heterozygous genotype individuals (Figure 7b–c), which exhibited low trans‐Atlantic differentiation relative to divergence by rearrangement genotype. Genetic distances between these clusters were larger in LG7 relative to LG2, indicating different rates or of differentiation.

In LG12, we observed low divergence among rearranged genotype individuals, suggesting this region is also shared across the North Atlantic (Figure 7d). We found strong separation between North America and Europe in nonrearranged genotype individuals in LG12 and observed distinct heterozygous genotype clusters for these regions, reflecting divergence in nonrearranged LG12 genotype individuals (Figure 7d).

Comparison of differentiation between North American and European individuals at the same rearranged genomic region revealed patterns consistent with adaptation from shared variation for each rearranged region, but also revealed elevated trans‐Atlantic divergence within nonrearranged haplotypes for LG1 and LG12 (Figure 7e,g,h). These values were significantly below the chromosome‐wide average for rearranged genotype individuals in LG1 (*W* = 5,129, *p* < .04, *F*_ST_ = 0.0184 rearranged region, *F*_ST_ = 0.0516 collinear region) and rearranged genotype individuals in LG12 (*W* = 13,332, *p* < 1.3 × 10^–7^, *F*_ST_ = 0.0328 rearranged region, *F*_ST_ = 0.0835 collinear region), but were not significantly different in LG2. Nonrearranged genotype individuals also exhibited reduced *F*_ST_ in the structurally variable region of LG7 (*W* = 5,975, *p* < .001, *F*_ST_ = 0.0464 rearranged region, *F*_ST_ = 0.0563 collinear region). In contrast, LG12 nonrearranged genotype individuals (*W* = 4,063, *p* < 2.2 × 10^–16^, *F*_ST_ = 0.505 rearranged region, *F*_ST_ = 0.084 collinear region Figure 7d) and LG1 nonrearranged genotype individuals (*W* = 8,966.5, *p* < 3.1 × 10^–13^, FST = 0.191 rearranged region, FST = 0.063 collinear region exhibited significantly elevated differentiation between North America and Europe. *F*_ST_ was consistently high at rearranged regions relative to regions without rearrangements (Figure S8). We found that *d_XY_* also tracked the same pattern as *F*_ST_ (Figure S9a–d).

Observed nucleotide diversity (*π*) values differed between rearranged and nonrearranged groups on LG1; rearranged genotype individuals in Europe (*W* = 192, *p* < 3.2 × 10^–5^) and North America (*W* = 189, *p* < 5.61 × 10^–6^) had lower nucleotide diversity than the remainder of the chromosome (Figure S9e–f). We found that *π* was reduced in structurally variable regions on LG12 for both rearranged genotype and nonrearranged genotype individuals in Europe (nonrearranged: *W* = 163, *p*‐value = .002078, rearranged: *W* = 191, *p* < 9.5 × 10^–7^) and North America (nonrearranged: *W* = 153, *p*‐value < .011, rearranged: *W* = 166, *p* < .0012).

## 4 DISCUSSION

Genomic architecture and standing variation can be crucial in determining evolutionary trajectories, particularly for instances of parallel evolution (Bolnick et al., 2018; Elmer & Meyer, 2011). In fact, genomic features may represent intrinsic drivers of adaptation and speciation, but their roles in this process have been underappreciated until recently (Campbell, Poelstra, & Yoder, 2018). Here, we investigated the extent of functional modularity and genomic parallelism in rearranged genomic regions in Atlantic cod and demonstrate that all four chromosomal rearrangements show low interchromosomal linkage within geographic regions, indicating these rearrangements exhibit independent inheritance. Further, our results support signatures of adaptation from shared variation in at least one chromosomal orientation in LG1, LG7, and LG12 chromosomal rearrangements, emphasizing the importance of shared architectural diversity. Interestingly, one chromosomal orientation for each of LG1 and LG12 has undergone trans‐Atlantic differentiation, suggesting nonparallel genomic adaptation for each of these regions. Together, these results demonstrate the importance of modular genomic architecture and both shared and nonparallel components of structural variation in enabling greater differentiation within Atlantic cod than previously recognized.

We demonstrate low interchromosomal linkage of rearranged regions, suggesting independent evolutionary trajectories for each rearrangement. These results are contrary to previous findings of high interchromosomal linkage between rearranged regions (Bradbury et al., 2014) and provide evidence against a genome‐wide barrier to reproduction. Our comparison of rearrangement genotypes within geographic regions revealed that previous observed patterns of interchromosomal linkage likely represent differences in rearrangement frequencies between regions. Widespread individual variation identified in our study also highlights potential genomic mechanisms underlying the presence of sympatric ecotypes occurring across the species range. Our observation of regional and individual levels of genetic structure associated with chromosomal rearrangements is consistent with the finding of physiological, behavioral, and life history variation within multiple discrete cod stocks, and observations of genetic divergence occurring within individual geographic regions (Conroy, Calvert, Sherwood, & Grabowski, 2017; Knutsen et al., 2018; Olsen et al., 2008; Puncher et al., 2019; Roney et al., 2016; Sinclair‐Waters, Bentzen, et al., 2018). Our identification of extensive individual‐level variation and low linkage in rearrangement genotypes indicates a genetic mechanism underpinning this biocomplexity: Atlantic cod distribution across differing environments may be enabled by locally adapted stocks within geographic regions, each with ecotype and individual variation in chromosomal rearrangement genotype. Independent inheritance of adaptive chromosomal rearrangements could facilitate exchange of these rearrangements as distinct adaptive units, enabling rapid production of novel ecotypes and colonization of a variety of habitats (Kirkpatrick & Barrett, 2015).

We found a shared pattern of migratory association of the LG1 rearrangement in independent polygenic association tests in Europe and North America. Associations of LG1 with migratory phenotype found here are consistent with past genome scan studies using migratory and nonmigratory ecotypes from waters around Europe (Berg et al., 2017) and genome‐wide association in North American waters (Kess et al., 2019). By using available migratory information for each site, we provide further support for the association of the LG1 rearrangement with migratory phenotype in the Eastern Atlantic. Recent molecular dating of this rearrangement indicates that its evolution substantially predates (1.6–2 Mya) postglacial colonization of Northwest Atlantic waters (Kirubakaran et al., 2016) and further supports the hypothesis of ancestral introduction of this rearrangement during colonization of North American waters (Sinclair‐Waters, Bentzen, et al., 2018).

Consistent with past studies using single‐locus association methods, we also find polygenic association of rearrangements with temperature, salinity, and oxygen availability in North America and Europe (Barney, Munkholm, Walt, & Palumbi, 2017; Berg et al., 2016; Bradbury et al., 2010). Although parallel clinal association is ecologically informative, autocorrelation of environmental variables within each site complicates interpretation of the exact adaptive role of each rearrangement (Hoban et al., 2016). We find associations for rearrangement frequencies with oxygen and salinity differed in direction and significance between North America and Europe, whereas the relationship with temperature and rearrangement frequency was significant and in the same direction on both continents, suggesting that temperature may primarily dictate clinal structuring of rearrangements.

Using multivariate co‐association and clustering, we show that SNPs within each rearrangement exhibited similar patterns of environmental co‐association. However, some SNPs overlapped in environmental associations between the rearrangements, suggesting a subset of loci are adapted to a shared set of environmental variables across rearrangements. This observation is consistent with these SNPs exhibiting functional modularity despite physical linkage. A similar pattern has also been observed among environment‐adapted genomic regions in *Pinus contorta*, indicating physical separation among loci is not always necessary for adaptation to different environmental variables (Lotterhos et al., 2018). Functional modularity was greater in Europe than in North America; our identification of divergence between the same structurally variable chromosome regions in Europe and North America likely contributes to varying patterns of environmental association identified here. Future studies using common garden experiments and crosses with manipulation of individual environmental parameters will be required to more clearly identify the functions of these structural variants (Bierne, Bonhomme, Boudry, Szulkin, & David, 2006; Wang et al., 2018).

We find signatures of adaptation from shared variation across the Atlantic for chromosomal orientations specific to each chromosome. Using differentiation‐based and genetic distance comparisons, we show low trans‐Atlantic differentiation between the rearranged orientation of LG1, and LG12, and the nonrearranged orientation of LG7, suggesting selection on shared variation in these regions in both Europe and North America. These patterns of localized reduced differentiation are consistent with incorporation of these genomic regions into the genetic architecture of adaptive traits from shared variation (Barrett & Schluter, 2008). This observation is similar to differentiation observed between marine and freshwater stickleback in comparison with the inverted region housing the *Atp1a1* gene, although the stickleback study focused specifically on multiple derived populations (Roesti, Gavrilets, Hendry, Salzburger, & Berner, 2014). Our results provide further support for the increasingly prominent role of shared structural variation in facilitating local adaptation (Barrett & Schluter, 2008; Kirkpatrick & Barton 2006).

We also uncovered elevated trans‐Atlantic differentiation in the nonrearranged orientations of LG1 and LG12, indicating distinct evolutionary histories of these chromosomal regions in Europe and North America. The identification of elevated trans‐Atlantic divergence within rearranged LG1 and LG12 genotype individuals suggests gene content in these regions may be amenable to repeated selection during ecological specialization in Atlantic cod. Repeated divergence of the same genomic region has been found to underlie parallel evolution of pelvic spine reduction in threespine stickleback through repeated deletions within *Pitx1* regulatory regions (Chan et al., 2010), and evolution of albinism in *Astyanax* cavefish has also arisen from independent deletions within the *Oca2* locus (Protas et al., 2006). Alternatively, reduced recombination and a long period of trans‐Atlantic divergence in ancient rearrangements predating speciation may also drive this elevated divergence relative to the genomic background. Surprisingly, we find that nucleotide diversity is reduced in the LG12 rearranged region for nonrearranged individuals despite high trans‐Atlantic *F*_ST_ and *d_XY_*, suggesting diversity‐reducing sweeps in these regions. However, low precision due to low density of SNPs, ascertainment bias, and high variance in *d_XY_* when applied to SNP data may bias measures of diversity and divergence (Wakeley, 1996). Future characterization of long‐read sequence from LG1 and LG12 across Europe and North American Atlantic cod may help clarify factors driving divergence identified here.

Shared, modular, structural variation is hypothesized to be integral to ecological adaptation and may facilitate repeatability of the genomic architecture underlying parallel adaptation. We show that modular, shared chromosomal rearrangements are associated with ecological adaptation within Atlantic cod. Additionally, we show that separate divergence in LG12 and LG1 rearrangements has occurred in North American and European populations, revealing a nonparallel component to adaptation within the same, structurally variable region of the genome. Our findings here also provide new understanding of biocomplexity organized at different spatial scales within this species. Substantial cryptic variation is mediated by these rearrangements, indicating different adaptive potentials among cod stocks. Understanding and conserving this variation may be crucial in mitigating anthropogenic impacts on Atlantic cod such as those imposed by climate change and overharvest.

## CONFLICT OF INTEREST

None declared.

## AUTHOR CONTRIBUTIONS

TK, SJL, IRB, and PB designed the study. TK, EVAS, and RF carried out statistical analyses. PB, IRB, MS‐W, MPK, and SL provided the molecular data. TK drafted the manuscript, and all authors contributed to writing and editing of the final manuscript.

## Data Availability

A plink format file of SNP genotypes for all genotyped individuals, lists of SNPs included in inversions, migratory phenotypes for European individuals, and environmental data for Europe and North America are available on dryad (https://doi.org/10.5061/dryad.f1vhhmgsg).
