# Supplementary figures and images for: Modular chromosome rearrangements reveal parallel and nonparallel adaptation in a marine fish
Source: Ecol Evol. 2020 Jan 11;10(2):638–53. doi: 10.1002/ece3.5828 (PMC6988541; doi:10.1002/ece3.5828)

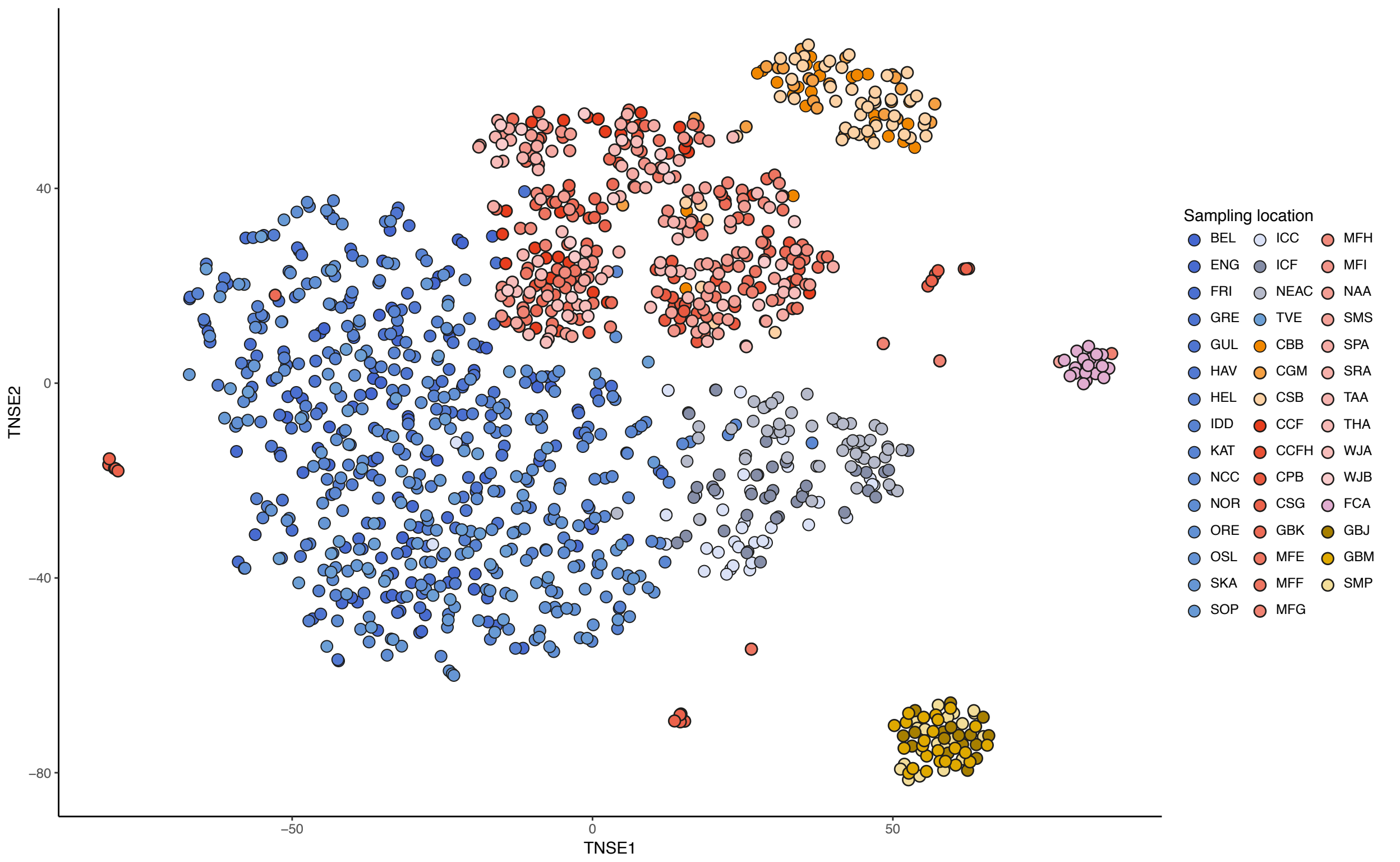

Supplement: Supplementary file 1 [file ECE3-10-638-s001.pdf]

**$-\log_{10}(\text{P value})$**

150

100

50

1

2

3

4

5

6

7

8

9

10

11

12

13

14

15

16

17

18

19

20

21

22

23

**Chromosome**

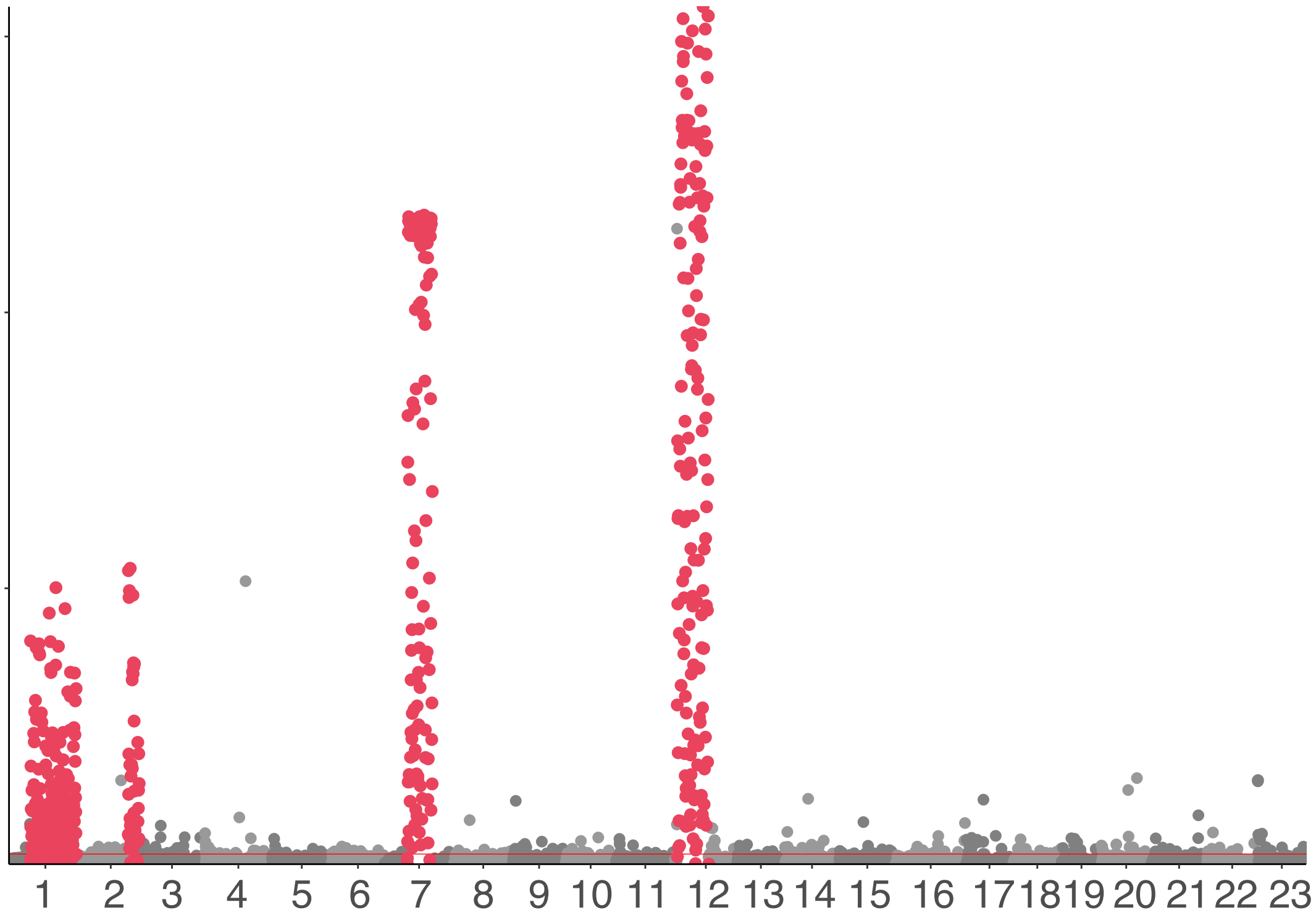

Supplement: Supplementary file 2 [file ECE3-10-638-s002.pdf]

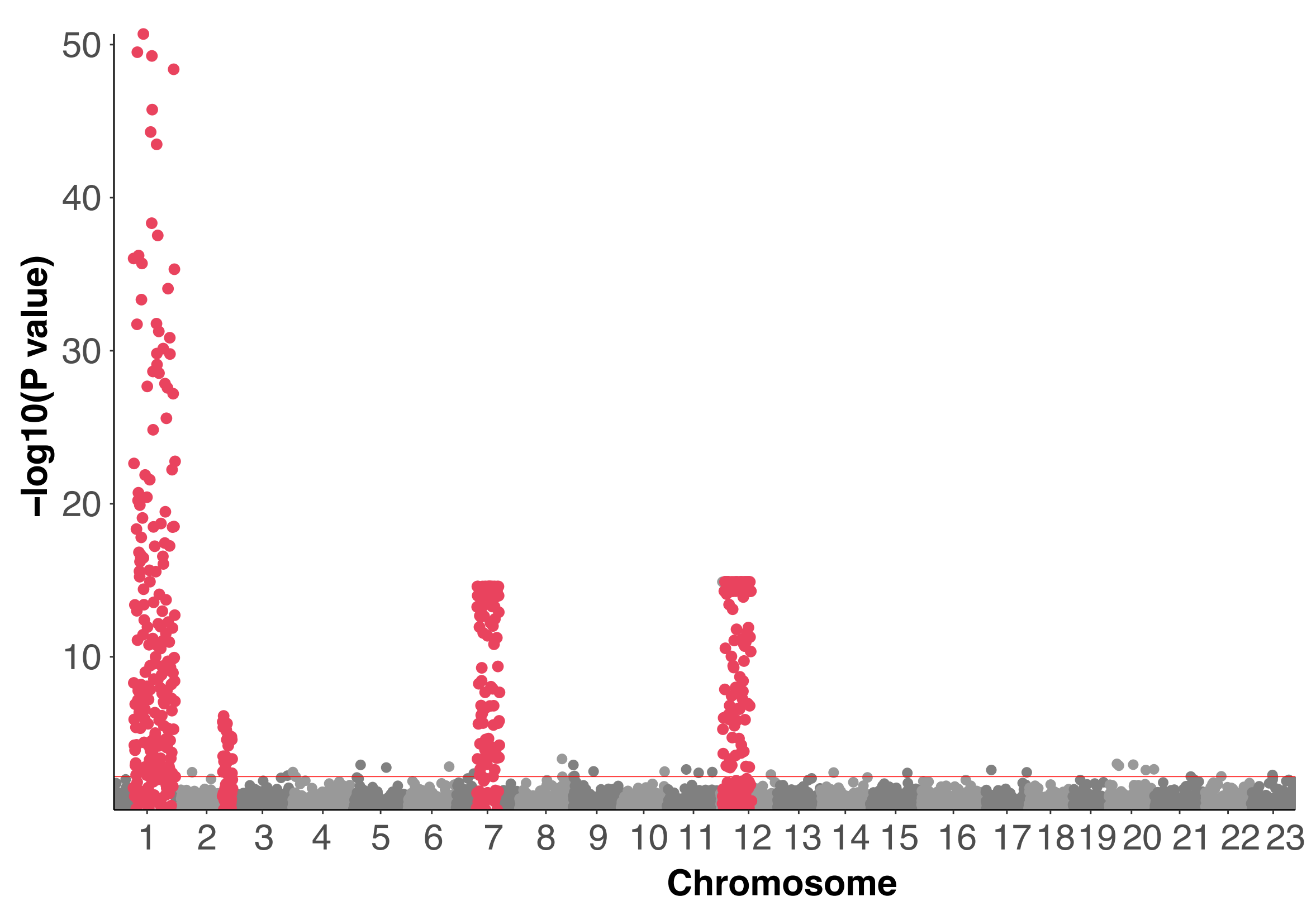

Supplement: Supplementary file 3 [file ECE3-10-638-s003.pdf]

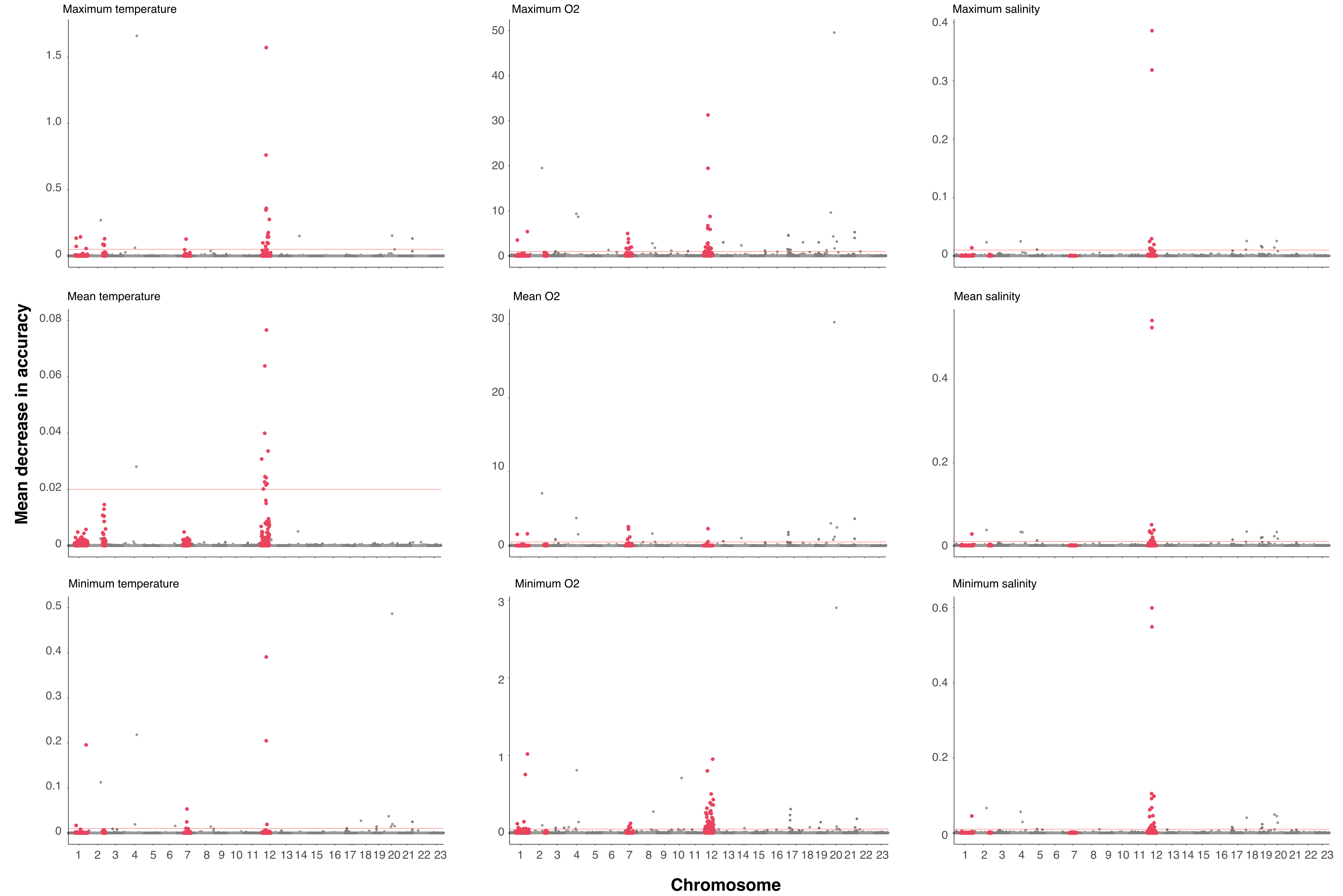

Supplement: Supplementary file 5 [file ECE3-10-638-s005.pdf]

Mean decrease in accuracy

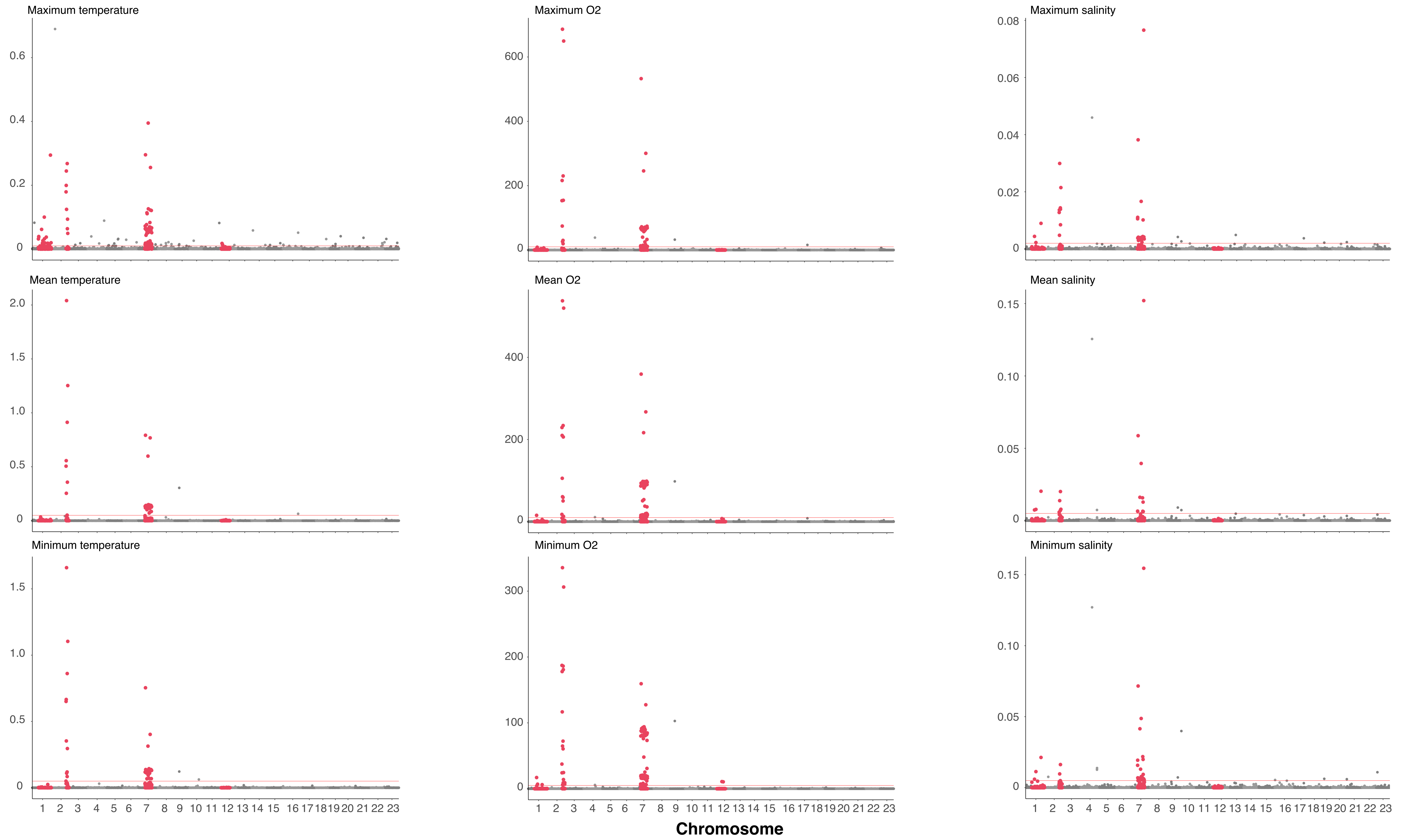

Supplement: Supplementary file 6 [file ECE3-10-638-s006.pdf]

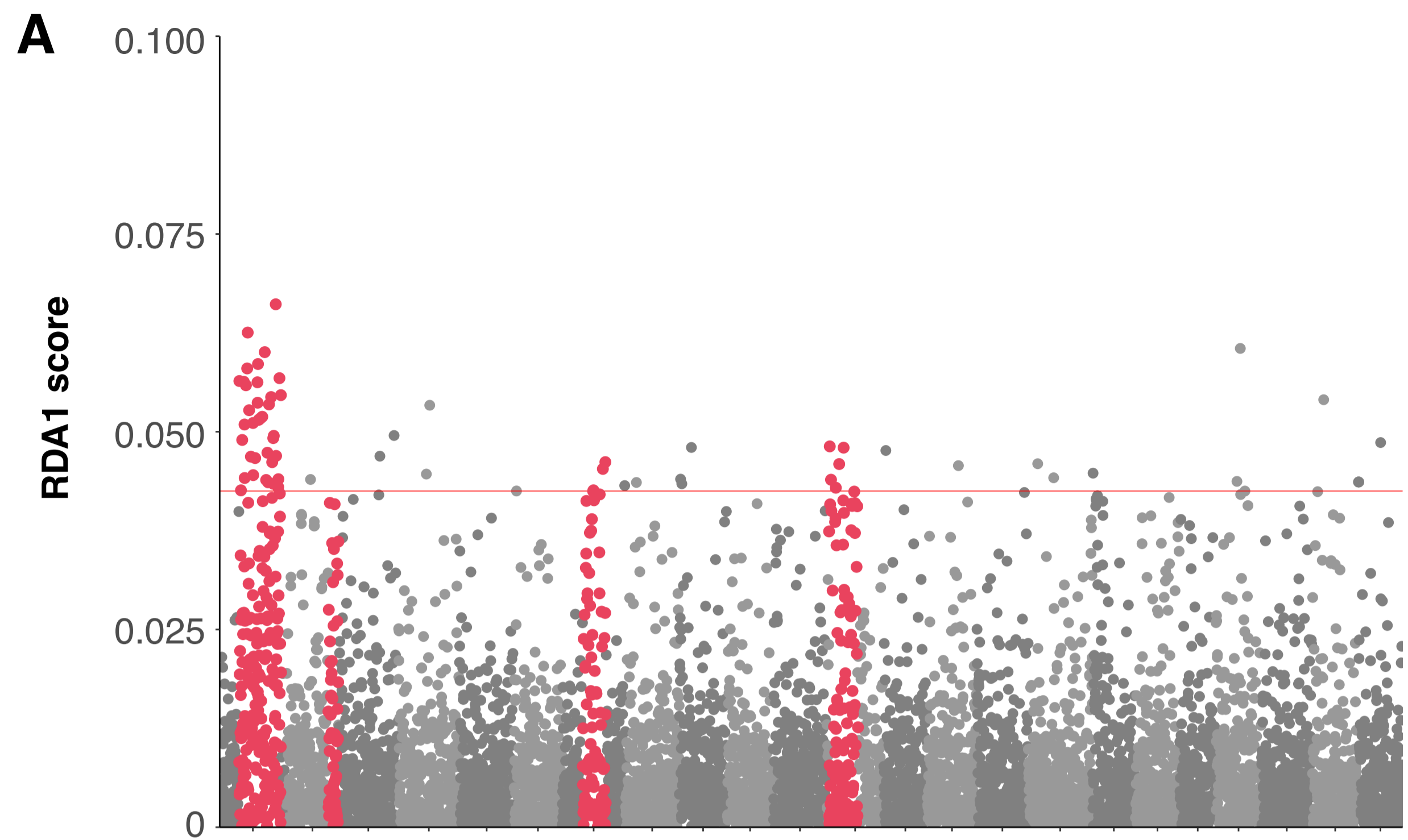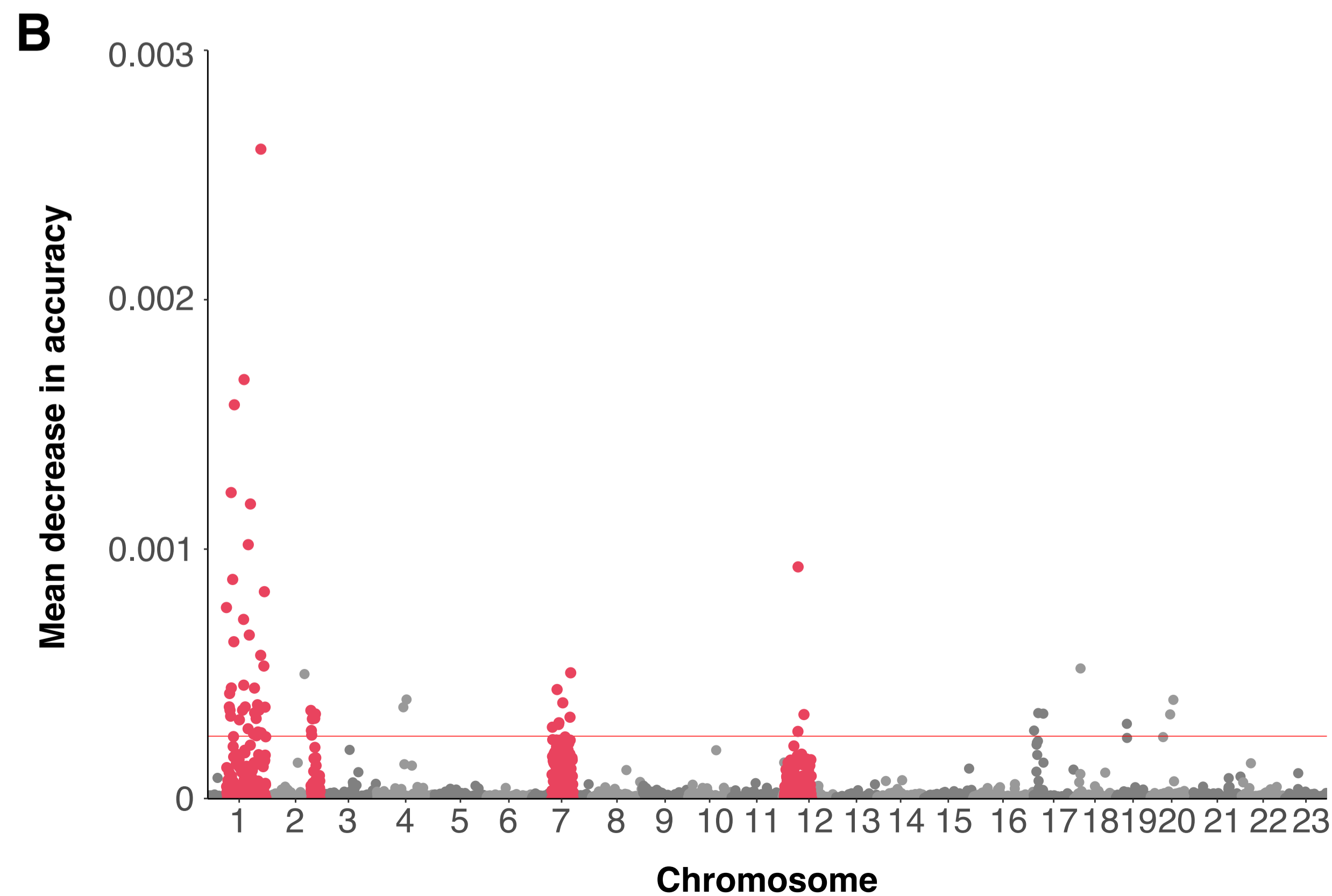

Supplement: Supplementary file 7 [file ECE3-10-638-s007.pdf]

$F_{ST}$

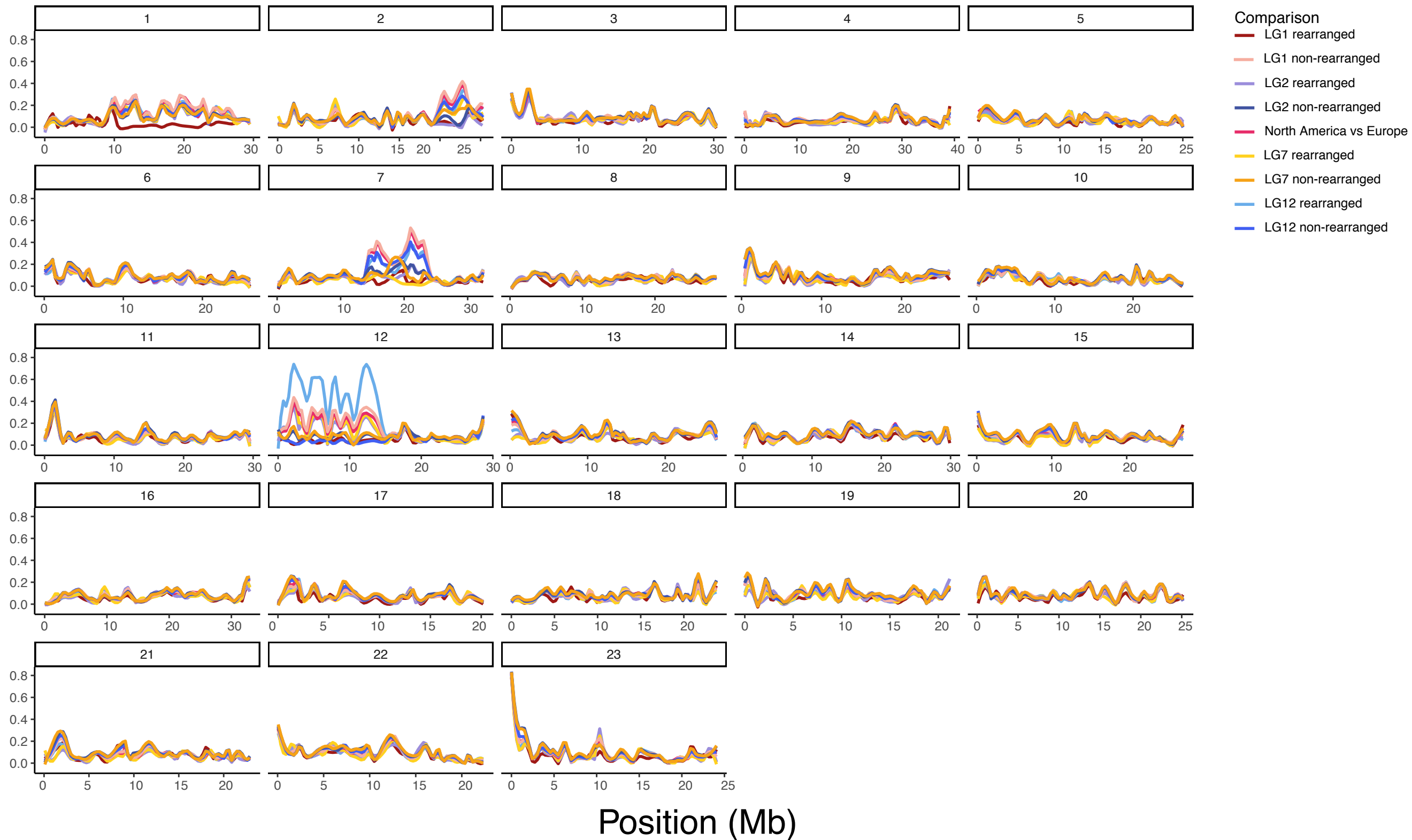

Supplement: Supplementary file 8 [file ECE3-10-638-s008.pdf]

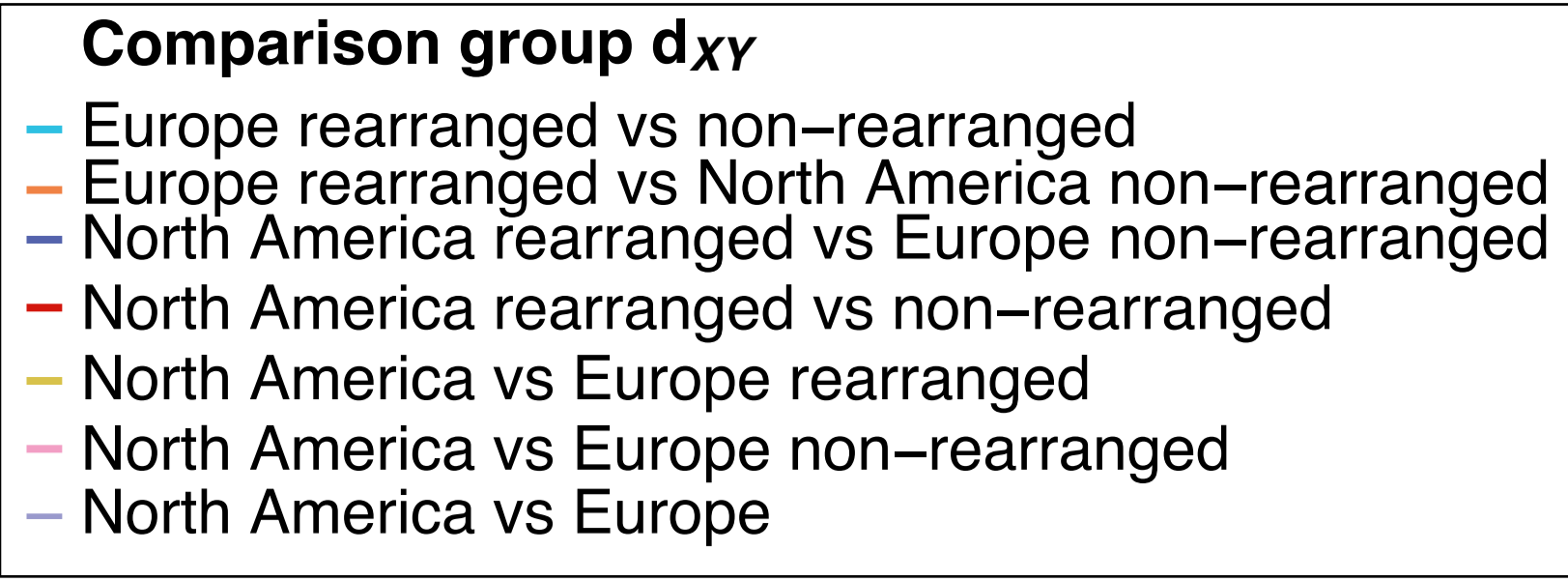

**A Rearrangement LG1**

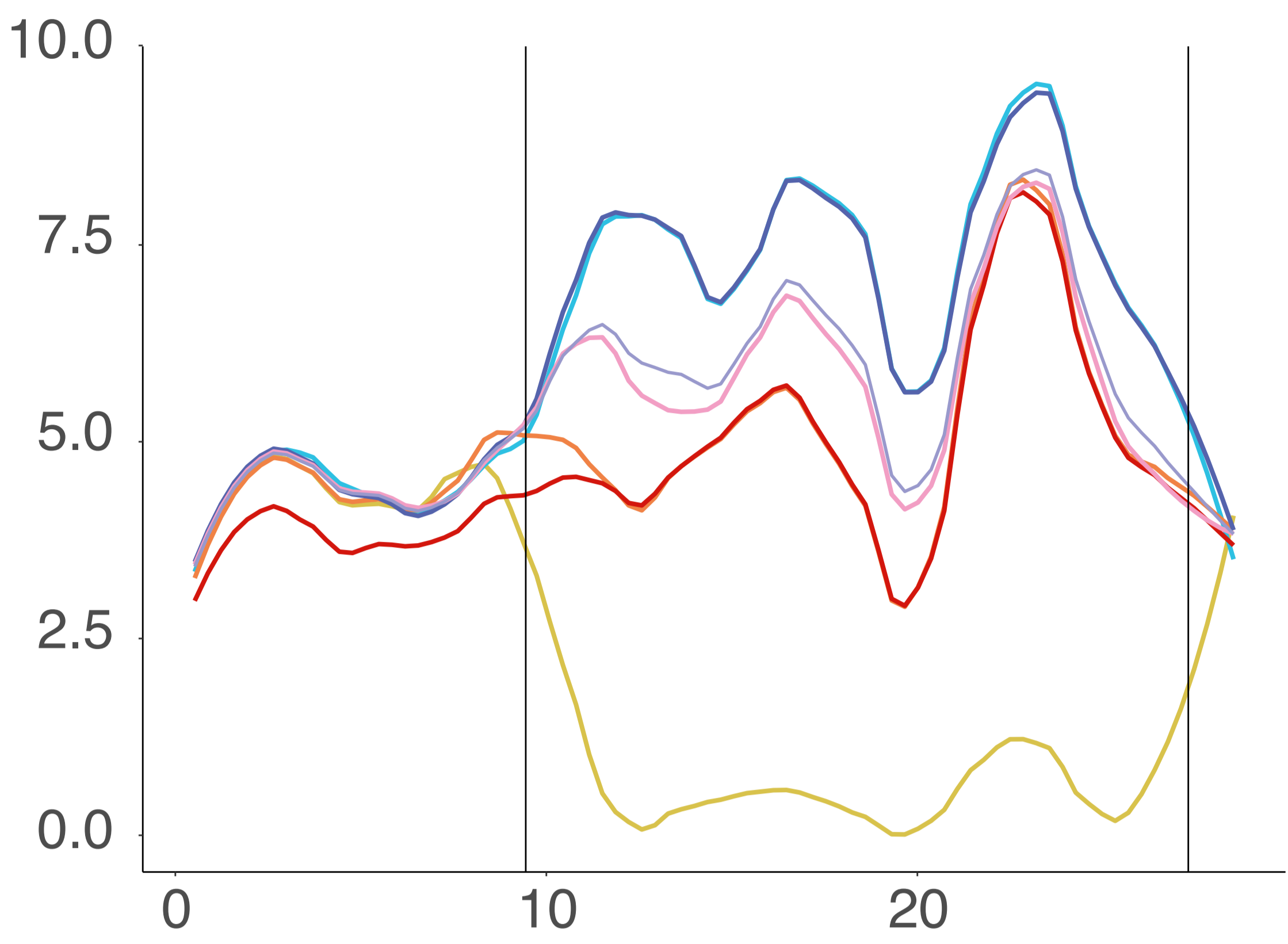

**B Rearrangement LG2**

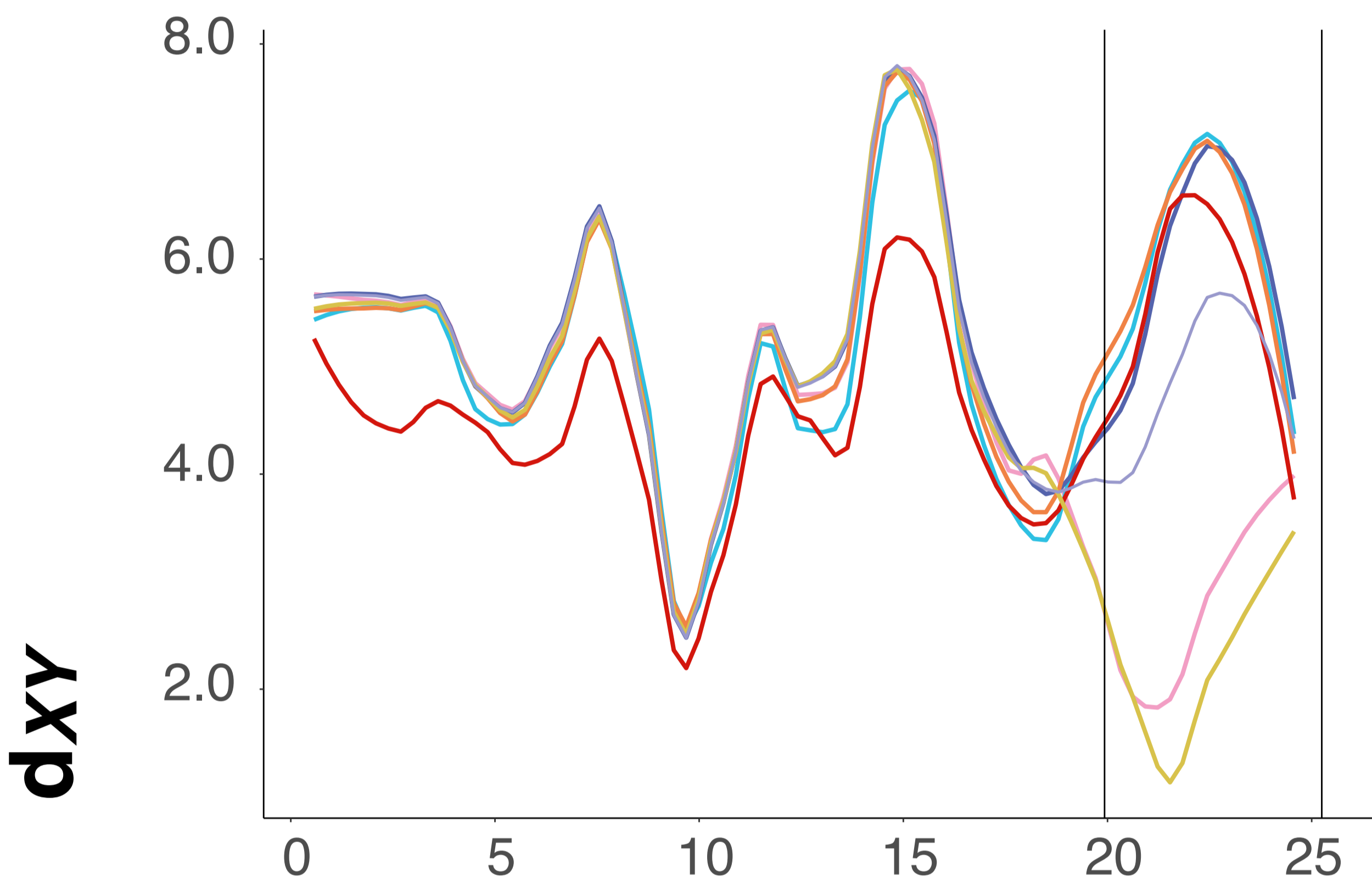

**C Rearrangement LG7**

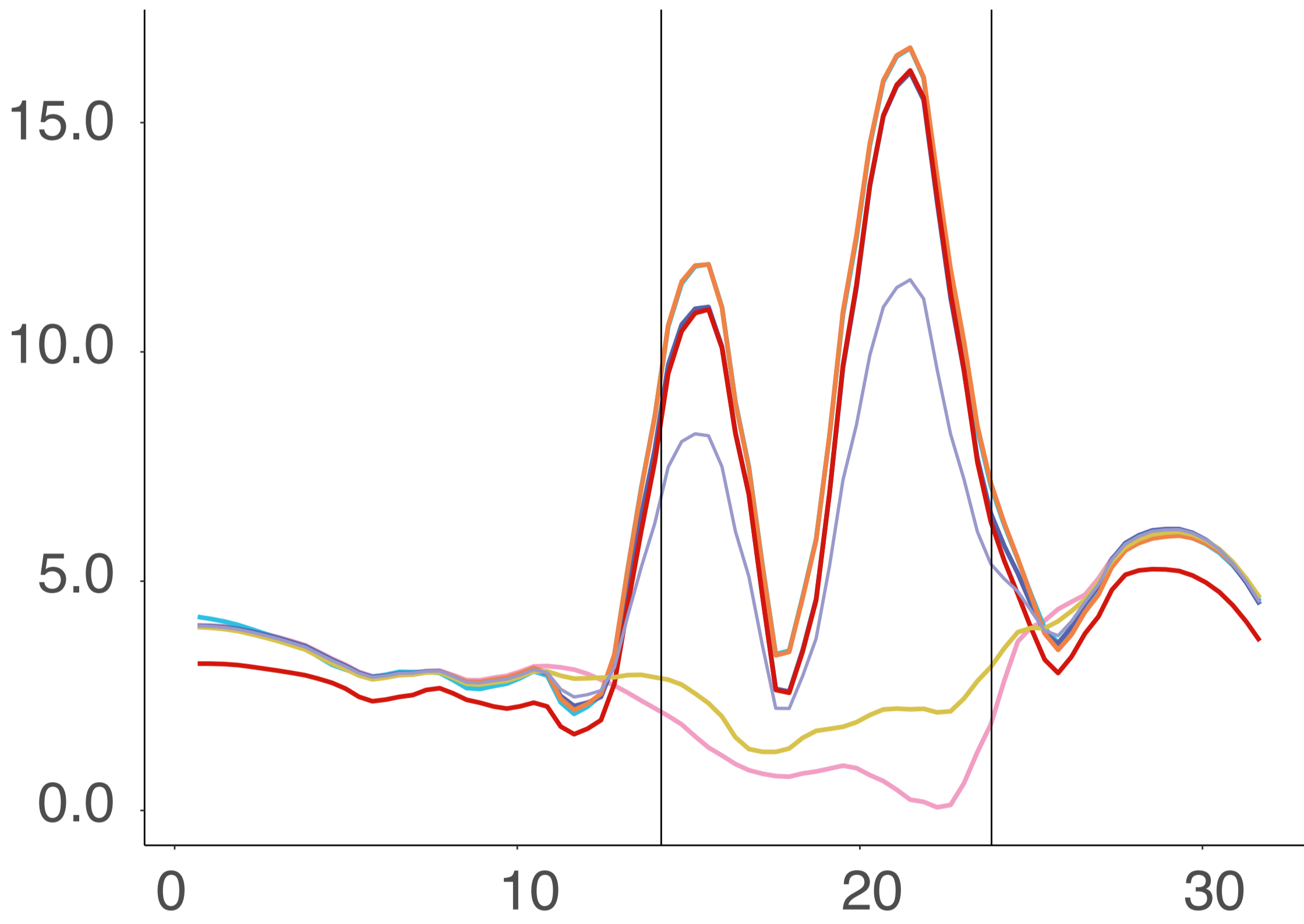

**D Rearrangement LG12**

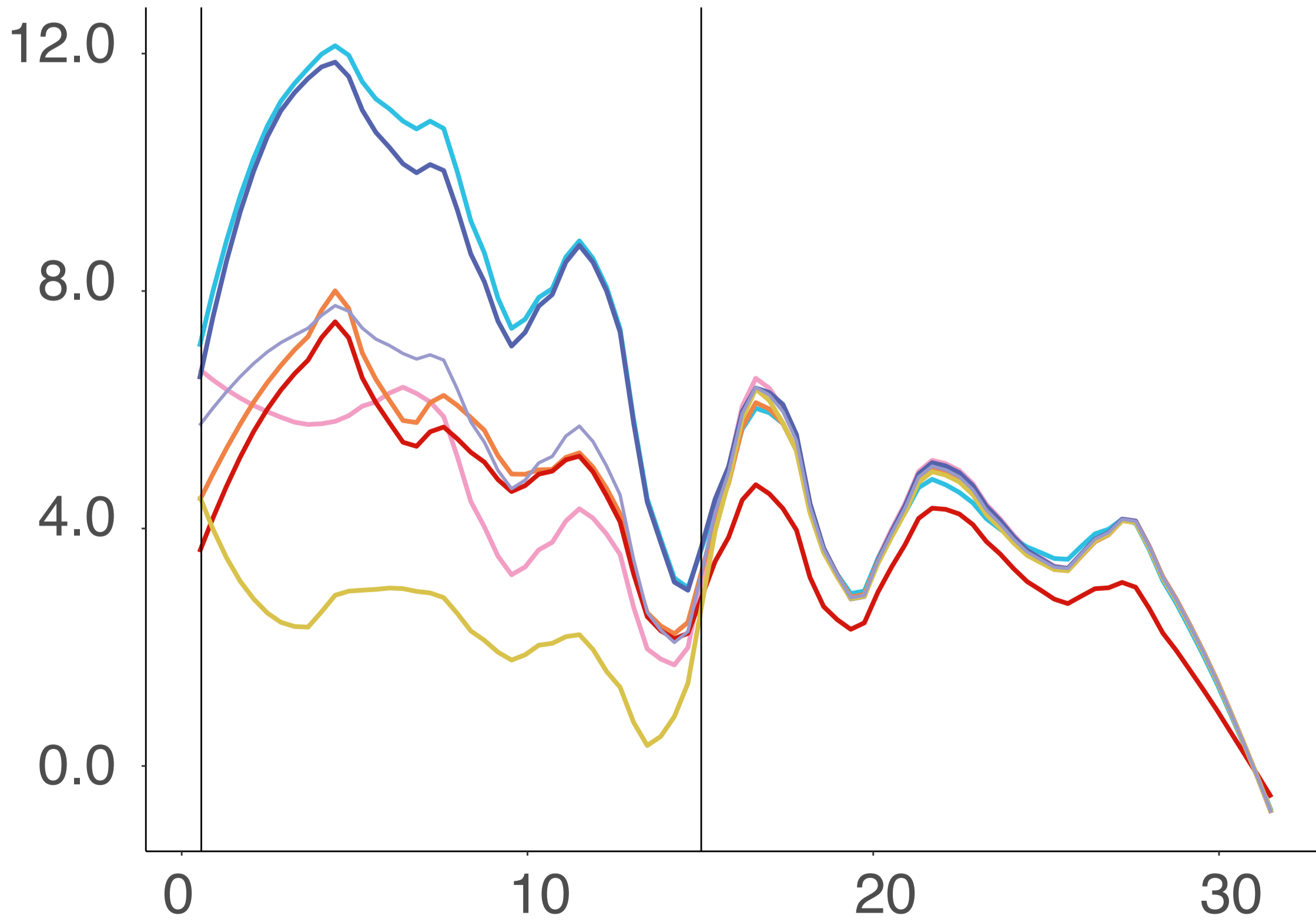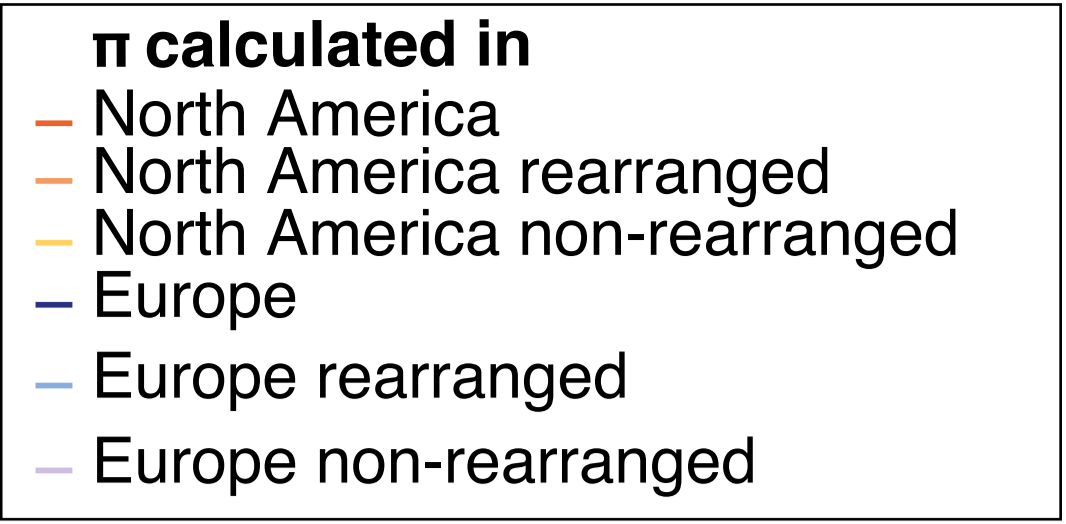

**E**

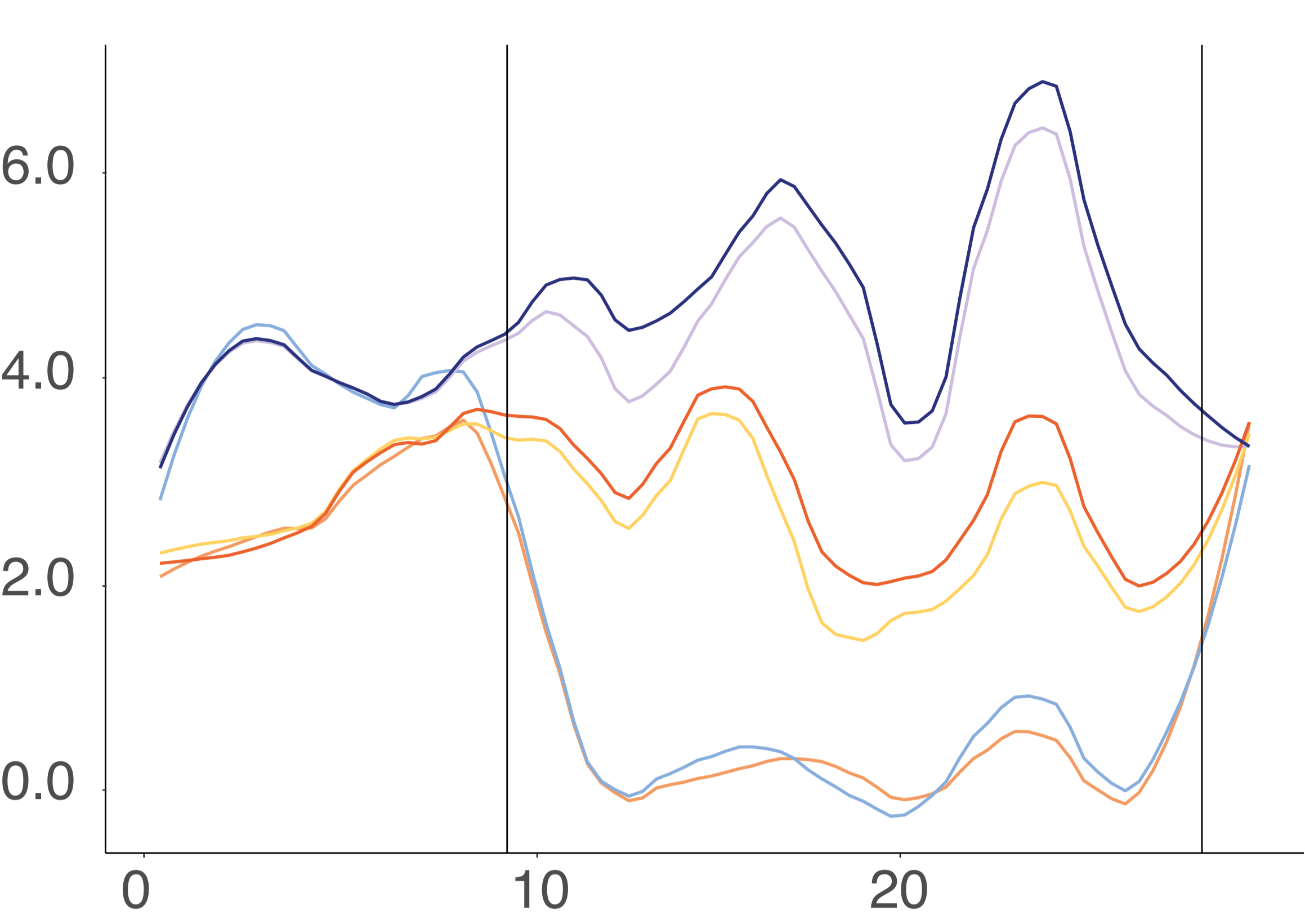

**F**

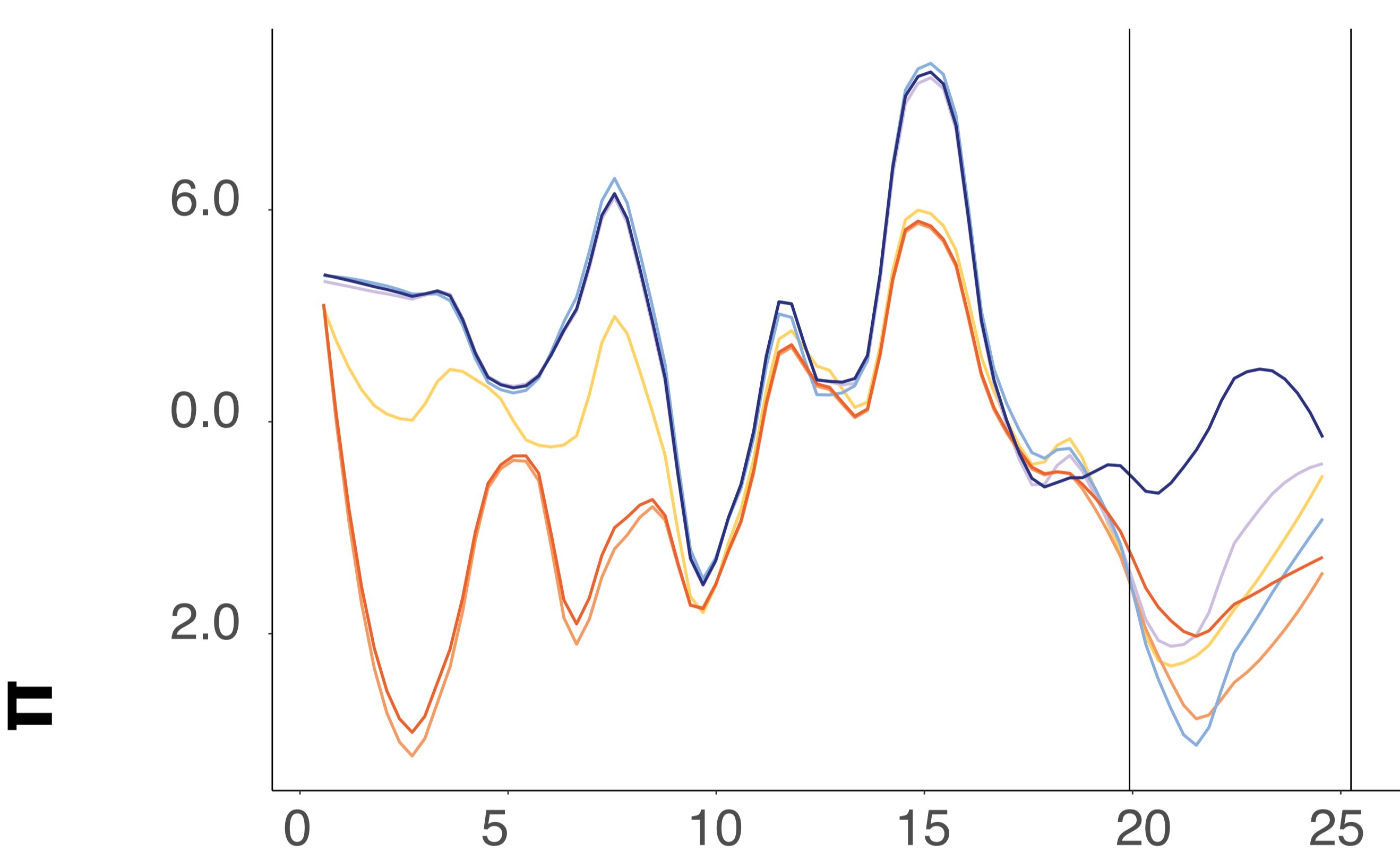

**G**

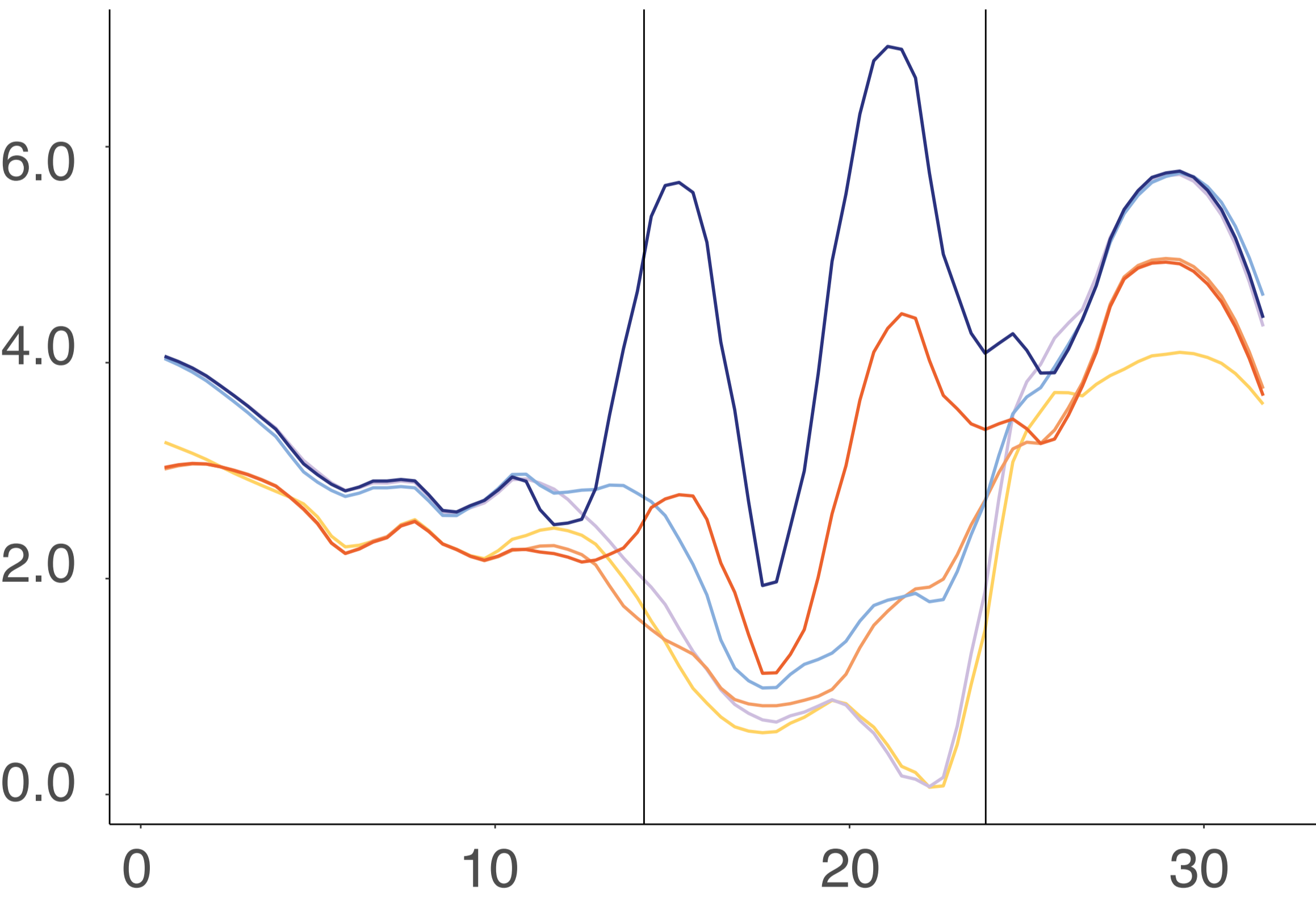

**H**

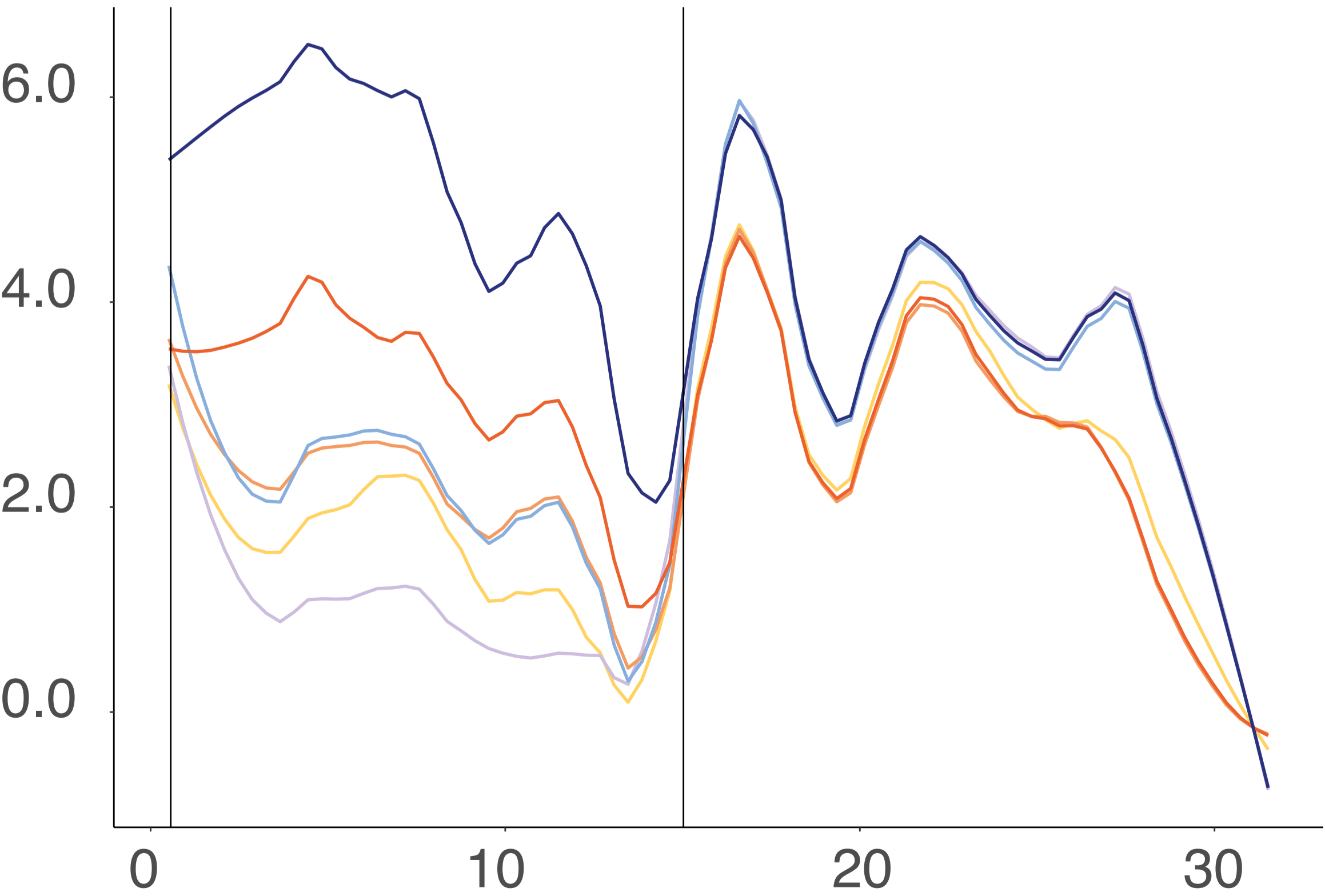

**Position (Mb)**

Supplement: Supplementary file 9 [file ECE3-10-638-s009.pdf]
