# Supplementary material for: Modular chromosome rearrangements reveal parallel and nonparallel adaptation in a marine fish
Source: Ecol Evol. 2020 Jan 11;10(2):638–53. doi: 10.1002/ece3.5828 (PMC6988541; doi:10.1002/ece3.5828)
Supplement: Supplementary file 4 [file ECE3-10-638-s004.pdf]

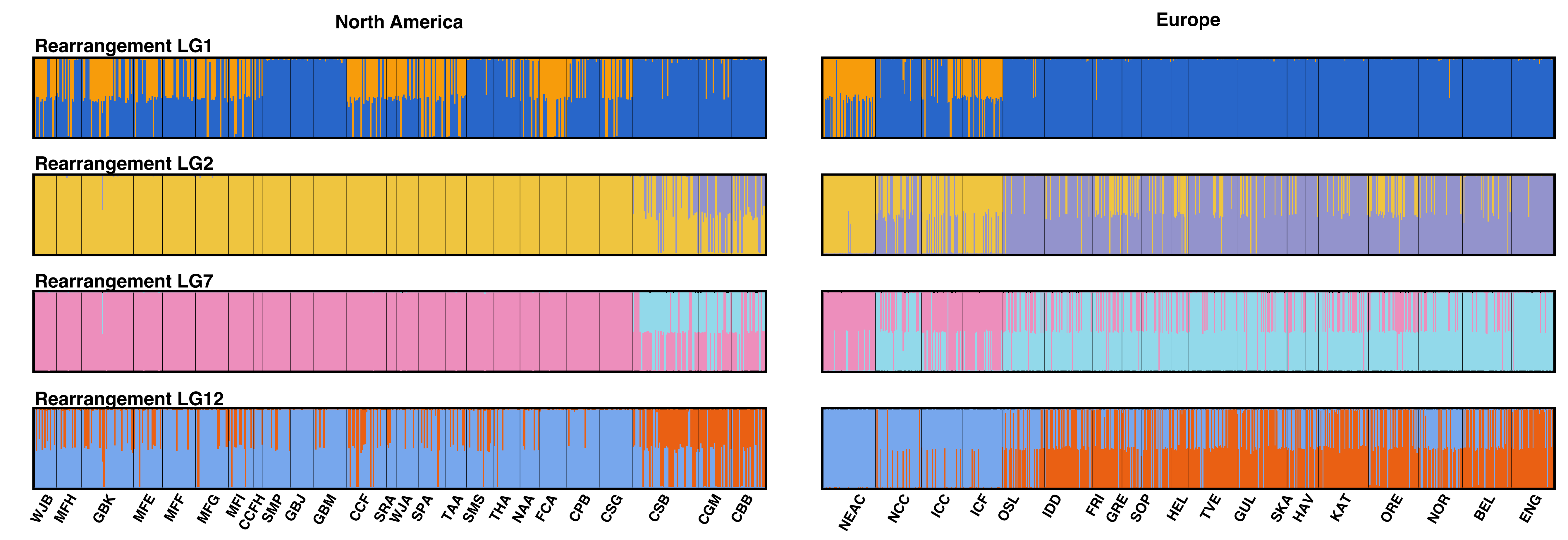
North America
Europe
Rearrangement LG1
Rearrangement LG2
Rearrangement LG7
Rearrangement LG12
WJB
MFH
GBK
MFE
MFF
MFG
MFI
CCFH
SMP
GBJ
GBM
CCF
SRA
WJA
SPA
TAA
SMS
THA
NAA
FCA
CPB
CSG
CSB
CGM
CBB
NEAC
NCC
ICC
ICF
OSL
IDD
FRI
GRE
SOP
HEL
TVE
GUL
SKA
HAV
KAT
ORE
NOR
BEL
ENG
